# Genomics-driven discovery of the pneumocandin biosynthetic gene cluster in the fungus *Glarea lozoyensis*

**DOI:** 10.1186/1471-2164-14-339

**Published:** 2013-05-20

**Authors:** Li Chen, Qun Yue, Xinyu Zhang, Meichun Xiang, Chengshu Wang, Shaojie Li, Yongsheng Che, Francisco Javier Ortiz-López, Gerald F Bills, Xingzhong Liu, Zhiqiang An

**Affiliations:** 1 State Key Laboratory of Mycology, Institute of Microbiology, Chinese Academy of Sciences, Beijing 100101, People’s Republic of China; 2 University of Chinese Academy of Sciences, Beijing 100049, People’s Republic of China; 3 Key Laboratory of Insect Developmental and Evolutionary Biology, Institute of Plant physiology and Ecology, Shanghai Institutes for Biological Sciences, Chinese Academy of Sciences, Shanghai 200032, People’s Republic of China; 4 Beijing Institute of Pharmacology & Toxicology, Beijing 100850, People’s Republic of China; 5 Fundación MEDINA, Centro de Excelencia en Investigación de Medicamentos Innovadores en Andalucía, Granada 18100, Spain; 6 Texas Therapeutics Institute, the Brown Foundation Institute of Molecular Medicine, University of Texas Health Science Center at Houston, Houston, TX 77030, USA

## Abstract

**Background:**

The antifungal therapy caspofungin is a semi-synthetic derivative of pneumocandin B_0_, a lipohexapeptide produced by the fungus *Glarea lozoyensis*, and was the first member of the echinocandin class approved for human therapy. The nonribosomal peptide synthetase (NRPS)-polyketide synthases (PKS) gene cluster responsible for pneumocandin biosynthesis from *G. lozoyensis* has not been elucidated to date. In this study, we report the elucidation of the pneumocandin biosynthetic gene cluster by whole genome sequencing of the *G. lozoyensis* wild-type strain ATCC 20868.

**Results:**

The pneumocandin biosynthetic gene cluster contains a NRPS (GLNRPS4) and a PKS (GLPKS4) arranged in tandem, two cytochrome P450 monooxygenases, seven other modifying enzymes, and genes for L-homotyrosine biosynthesis, a component of the peptide core. Thus, the pneumocandin biosynthetic gene cluster is significantly more autonomous and organized than that of the recently characterized echinocandin B gene cluster. Disruption mutants of GLNRPS4 and GLPKS4 no longer produced the pneumocandins (A_0_ and B_0_), and the *Δglnrps4* and *Δglpks4* mutants lost antifungal activity against the human pathogenic fungus *Candida albicans*. In addition to pneumocandins, the *G. lozoyensis* genome encodes a rich repertoire of natural product-encoding genes including 24 PKSs, six NRPSs, five PKS-NRPS hybrids, two dimethylallyl tryptophan synthases, and 14 terpene synthases.

**Conclusions:**

Characterization of the gene cluster provides a blueprint for engineering new pneumocandin derivatives with improved pharmacological properties. Whole genome estimation of the secondary metabolite-encoding genes from *G. lozoyensis* provides yet another example of the huge potential for drug discovery from natural products from the fungal kingdom.

## Background

**Figure 1:**
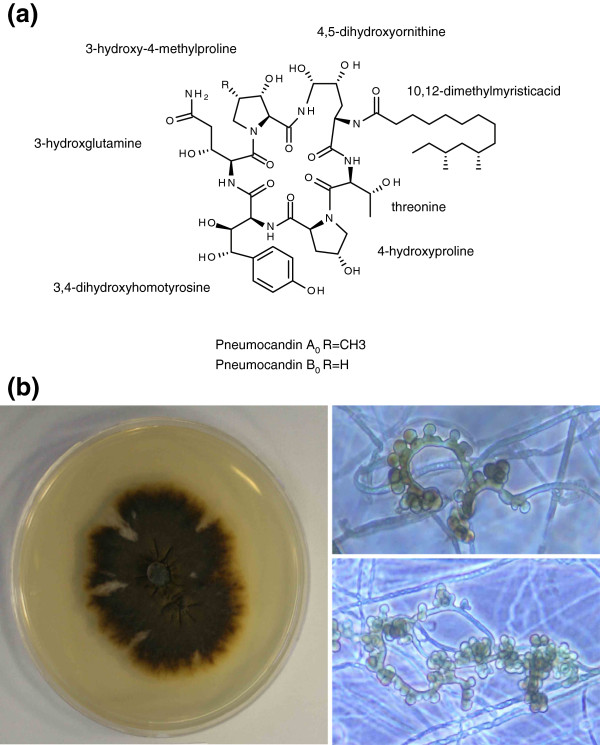
**Pneumocandin structures and morphology of *Glarea lozoyensis*.** (**a**) Chemical structures of pneumocandins. (**b**) Colony of *G. lozoyensis* on malt yeast agar (left panel); conidiophores and conidia of *G. lozoyensis* (right panels).

Fungi frequently cause deadly infections in immunocompromised patients resulting from HIV infection, cancer chemotherapy, and organ transplantation [1]. Until the introduction of caspofungin (CANCIDAS™) in 2001, antifungal therapy was limited to the use of polyenes (amphotericin B), azoles, and flucytosine which have high failure rates during management of fungal infection, while experiencing increasing clinical resistance [1]. The echinocandins are a class of antifungal lipopeptides targeting fungi via noncompetitive inhibition of the β-1,3-d-glucan synthase enzyme complex, leading to glucan polymer depletion in the fungal cell wall and resulting in osmotic instability and fungal cell lysis [1]. Human side effects to these chemicals are minimal because the target is absent in mammalian cells, and low dosing is used due to the drug’s potent efficacy [1,2]. Thus far, three echinocandin-based agents have been approved for clinical use [1]. Caspofungin, a semi-synthetic derivative of pneumocandin B_0_ (Figure 1a) which is a lipohexapeptide produced by the filamentous fungus *Glarea lozoyensis* (Figure 1b), was the first member of this class approved for human therapy; its registration was followed by micafungin (MYCAMINE™) derived from FR901370 (WF11899A), a sulfonated hexapeptide produced by the fungus *Coleophoma empetri*[3], and lastly anidulafungin (ERAXIS™) derived from echinocandin B produced by the fungus *Aspergillus rugulosus*[4]. The three fungal metabolites share a common chemical structure of cyclic lipohexapeptide with *N*-acylated to either 10,12-dimethylmyristoyl (pneumocandins) or palmitoyl (FR901370) or linoleoyl (echinocandin B); their hexapeptide cores differ from each other by modifications on 4-hydroxyproline or dihydroxyhomotyrosine (FR901370 and pneumocandins possess 3-hydroxyglutamine, while echinocandin B has threonine substituted in the same position) [5-8]. Because of their high efficacy, they have become the first-line therapy for the treatment of invasive fungal infections [1].

Several cases of *in vivo* caspofungin resistance have been reported for *Candida* and *Aspergillus* species caused by mutations that reduce the drug sensitivity of the glucan synthase by several thousand-fold [9-12]. A compensatory cell wall remodeling mechanism elevating the chitin content has been found to be associated with caspofungin resistance in *C. albicans*[13-15]. Generation of pneumocandin derivatives with more desirable pharmacological properties via medicinal chemistry approaches has proven difficult [16,17]. Elucidation of the biosynthetic pathway to pneumocandins is the first step in applying pathway manipulation and biocombinatorial chemistry approaches to engineer new derivatives with broader spectra of activity and improved physiochemical characteristics to meet the challenges of broader efficacy and clinical resistance.

Based on the structure of pneumocandin, participation of a nonribosomal peptide synthetase (NRPS) and a polyketide synthase (PKS) are predicted for biosynthesis of the cyclic hexapeptide and the 10,12-dimethylmyristoyl side chain [18,19], respectively. Previous attempts to clone the NRPS and PKS gene cluster responsible for pneumocandin biosynthesis from *G. lozoyensis* have been unsuccessful [20,21]. Whole genome sequencing has proven to be an efficient approach in the identification of gene clusters of fungal secondary metabolites, such as PKSs and NRPSs [22]. A recent genomic sequencing project of a pneumocandin B_0_-overproducing mutant (ATCC 74030) derived from the wild-type (WT) strain of *G. lozoyensis* was inconclusive in identifying the pneumocandin biosynthetic cluster due to insufficient genome coverage [23]. In this study, we report the elucidation of the pneumocandin biosynthetic gene cluster by genome sequencing of the *G. lozoyensis* WT strain ATCC 20868. We also compare gene cluster organization with that of the recently published echinocandin B biosynthetic cluster [8,24]. In addition, analysis of the *G. lozoyensis* genome revealed a rich repertoire of secondary metabolite-encoding genes that once again illustrates the huge potential for drug discovery from natural products from the fungal kingdom.

## Results

### The genome characteristics of *G. lozoyensis*

**Figure 2:**
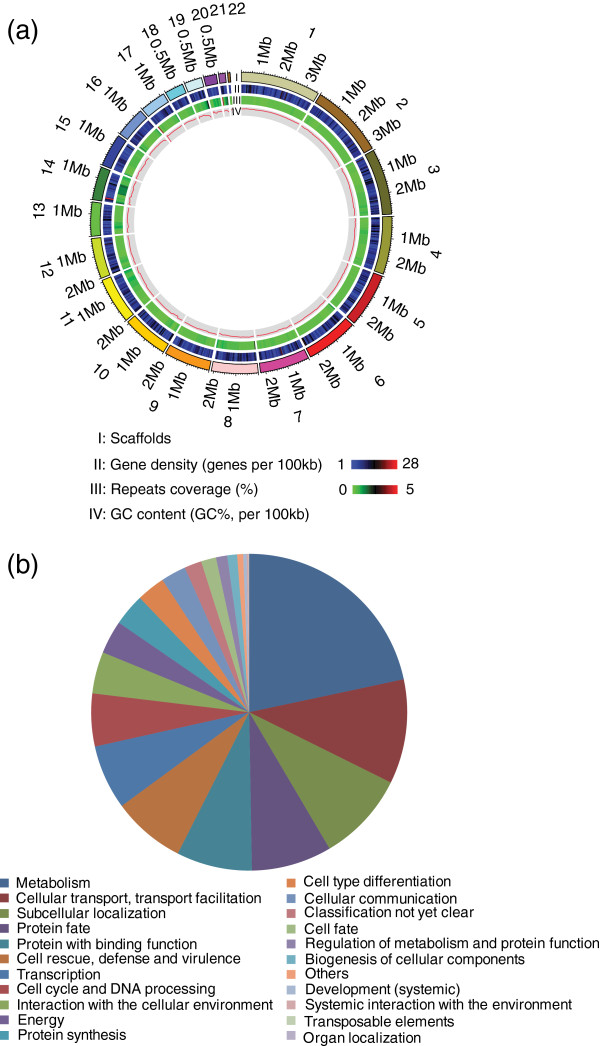
**Genome features of *Glarea lozoyensis*.** (**a**) General genome features of *G. lozoyensis*. I, 22 scaffolds (> 2 kb); II, gene density represented as number of genes per 100 kb; III, percentage of coverage of repetitive sequences; IV, GC content was estimated by the percent G + C in 100 kb. (**b**) Functional classificaton of proteins in the *G. lozoyensis* genome based on InterproScan analysis.

**Figure 3:**
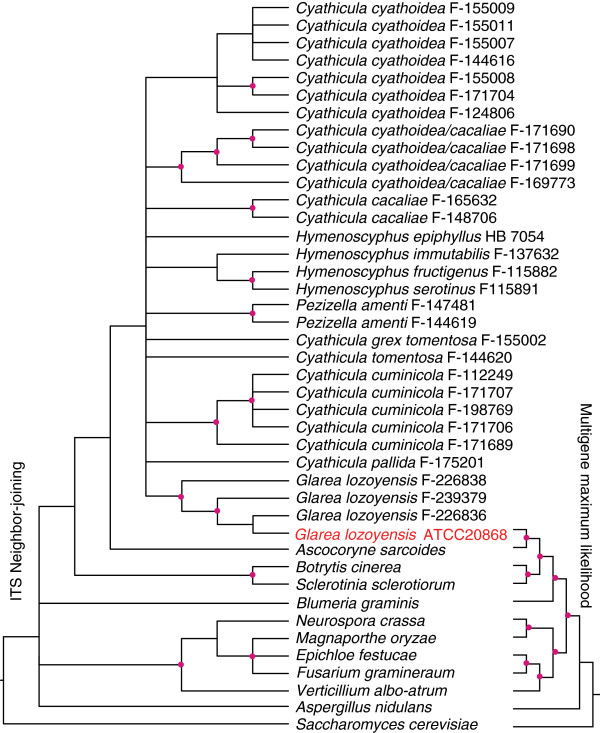
**Phylogenetic analysis of *G. lozoyensis *using ITS sequences (brackets on the left) and genome protein sequences (brackets on the right).** Left tree: The topology was estimated using neighbor-joining method based on the ITS sequence data from Peláez et al., 2011 [26] and the selected fungi on the right-side of the graphic. Right tree: A maximum likelihood phylogenomic tree showing evolutionary relationship of *G. lozoyensis* with selected ascomycete fungal species. The tree was constructed from the concatenated amino acid sequences of 878 common orthologous genes (Additional file 2: Table S2). The phylogenetic position of *G. lozoyensis* wild-type strain ATCC 20868 is marked in red. Branch nodes with greater than 60% support from 1000 bootstrapped pseudoreplicates are indicted with red dots in both trees. Both trees were rooted with *S. cerevisiae*.

**Figure 4:**
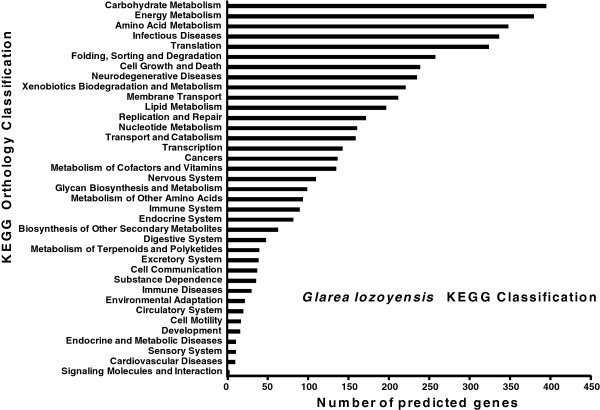
**KEGG functional classification of proteins in the *G. lozoyensis *genome.** Distribution of the predicted proteins were assigned by the Kyoto Encyclopedia of Genes and Genomes (KEGG) database. The top four categories in the KEGG functional classification were Carbon Metabolism, Energy Metabolism, Amino Acid Metabolism, and Infectious Diseases.

Sequencing of the *G. lozoyensis* WT strain ATCC 20868 with an 80× genome coverage revealed a high resolution 39.6-megabase (Mb) genome with 0.5% repeat content. Reads were assembled into 22 scaffolds (>2 kb; N_50_, 2.45 Mb) incorporating more than 99% of the total genomic base pairs (Figure 2a). The average gene density was 330 genes per Mb (Table 1). The 13,103 putative coding genes were assigned to different functional categories (Figure 2b). Consistent with previous studies by our group [25,26], a combined phylogenomic and phylogenetic analysis confirmed that *G. lozoyensis* belonged the same major phylogenetic lineage as the plant pathogenic fungi, *Sclerotinia sclerotiorum* and *Botrytis cinerea*[27], and the wood endophytic fungus, *Ascocoryne sarcoides*, the Helotiales [28] (Figure 3). A total of 4931 predicted proteins were assigned by the Kyoto Encyclopedia of Genes and Genomes (KEGG) database. The top four categories in the KEGG functional classification were “Carbon Metabolism, Energy Metabolism, Amino Acid Metabolism, and Infectious Diseases” (Figure 4).

**Table 1:** **General features of the *G. lozoyensis *genome**

**Features**	
Assembly size (Mb)	39.6
Scaffold N_50_ (kb)	2453
Coverage (fold)	80
G + C content (%)	45.8
GC exonic (%)	49.06
GC intronic (%)	41.98
Repeat rate (%)	0.5
Protein-coding genes	13103
Gene density (per Mb)	330.38
Exons per gene	2.98
tRNAs	131
rRNAs	22
NRPSs	6
PKSs	24
PKS-NRPS hybrids	5
DMATSs	2
Terpene synthases	14
NRPS-like	13
PKS-like	1
Chalcone or stilbene synthase gene	1
Secondary metabolite gene clusters	49

*Mb* mega bases, *PKS* polyketide synthase, *NRPS* nonribosomal peptide synthetase, *PKS-NRPS hybrid* polyketide synthase-nonribosomal peptide synthetase hybrid, *DMATS* dimethylallyl tryptophan synthase, *tRNAs* transfer RNA, *rRNA* ribosomal RNA.

**Figure 5:**
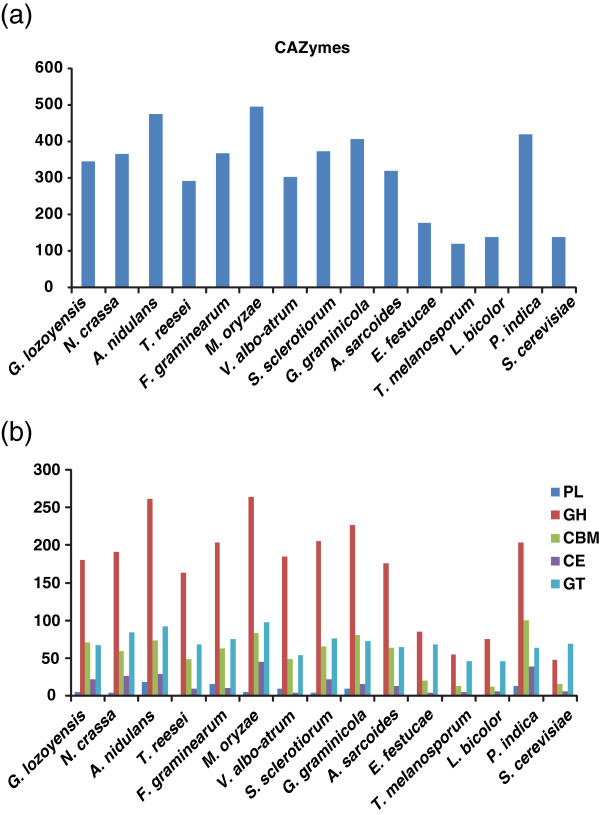
**CAZymes (carbohydrate-active enzymes) analysis in the *G. lozoyensis *genome and other fungi.** (**a**) Total number of CAZymes in different fungi (*Glarea lozoyensis*, *Neurospora crassa*, *Aspergillus nidulans, Trichoderma reesei, Fusarium graminearum, Aspergillus oryzae, Verticillium albo-atrum*, *Sclerotinia sclerotiorum*, *Glomerella graminicola*, *Ascocoryne sarcoides*, *Epichloë festucae*, *Tuber melanosporum*, *Laccaria bicolor*, *Piriformospora indica*, and *Saccharomyces cerevisiae*). (**b**) Number of different family of CAZymes in different fungi. PL: polysaccharide lyase, GH: glycoside hydrolase, CBM: carbohydrate-binding module, CE: carbohydrate esterases, and GT: glycosyltransferase. See Additional file 2: Table S2 for a detailed tabular summary.

Strains of *Glarea lozoyensis* have been isolated from water, plant litter or soil samples [25,26]. However, the fungus has never been observed in nature, therefore its ecological role and trophic relationships remain unknown. It has been speculated that the fungus may be a plant or plant litter saprobe for the following reasons [25,26]. The fungus belongs to the same phylogenetic lineage as *Cyathicula* or *Crocicreas*, an inconspicuous group of fungi that are weak parasites, endophytes of living plants or saprobes of senescent plants and plant litter. In the laboratory, the fungus readily colonized and sporulated on sterilized hardwood [25]. Its asexual sporulation (Figure 1b) resembled that of a heterogeneous group of asexually reproducing fungi known as aero-aquatic fungi that often colonize plant debris in periodically inundated habitats [29,30]. Several recent studies have demonstrated a strong relationship between the suite of carbohydrate active enzymes (CAZymes, http://www.cazy.org) in fungal genomes and their saprobic, parasitic or necrotrophic life strategies [31-33]. Such investigations have focused on those CAZymes involved in polysaccharide degradation and have contributed to a thorough understanding of the ecological role of a fungus. To infer whether *G. lozoyensis* might be a biotroph, saprotroph or necrotroph, we analyzed its complement of CAZy gene families and genes. The putative CAZymes in *G. lozoyensis* were identified using the CAZy annotation pipeline (http://mothra.ornl.gov/cgi-bin/cat.cgi) [34,35] and were compared to a selection of ascomycete and basidiomycete fungi (Figure 5a and 5b). At least 345 CAZymes in the five principal category families were identified in the genome (Figure 5a). This value is similar to the number of CAZymes found in known plant cell wall degrading ascomycetes, including the wood-inhabiting endophyte *A. sarcoides*, but significantly higher than the yeast *Saccharomyces cerevisiae*, and the plant biotrophic symbionts *Laccaria bicolor*, *Epichloë festucae*, and *Tuber melanosporum* (Figure 5a). A total of 180 glycoside hydrolases (GH) in 70 families were found in the *G. lozoyensis* genome, which is slightly less than average compared to other filamentous plant associated ascomycetes [36]. Likewise, the number of 67 glycosyl transferases (GT) in 35 families was also comparable to other plant inhabiting ascomycetes (Figure 5b). Average numbers of polysaccharide lyases (PL, 5), carbohydrate esterases (CE, 22) were found. However, a relatively abundant number of carbohydrate binding modules (CMB, 71) were identified. Therefore, its complement of genes associated with carbohydrate degradation and metabolism were consistent with those of other plant-associated ascomycetes.

### *G. lozoyensis* genome revealed a rich repertoire of secondary metabolite-encoding genes

To identify the pathways involved in the synthesis of secondary metabolites in *G. lozoyensis*, we searched the genome for genes encoding key enzymes such as NRPS, PKS, terpene synthase (TS), and dimethylallyl tryptophan synthase (DMATS), which are essential for the biosynthesis of peptides, polyketides, terpenes, and alkaloids, respectively. The following secondary metabolite-encoding genes were dispersed among 49 gene clusters: six NRPSs, 24 PKSs, five polyketide synthase-nonribosomal peptide synthase hybrids (PKS-NRPS hybrids), 14 TSs, two DMATSs, 13 NRPS-like, one PKS-like, and one chalcone/stilbene synthase gene (Table 1). In addition to genes encoding the core enzyme(s), the majority of the 49 secondary metabolism gene clusters in *G. lozoyensis* contained genes encoding other biosynthesis enzymes, transcription regulators, and transporters. For example, about half of the gene clusters contained a gene encoding a Zn_2_/Cys_6_ or a C_2_H_2_ and C_2_HC zinc transcriptional factor that could control the expression of genes within of its own cluster. Also, about 60% of the secondary metabolism clusters contained a gene encoding an ABC or a MFS transporter(s) that could export the metabolites produced by the enzymes encoded by the gene cluster (Additional file 1: Figure S1).

### Biosynthetic capabilities of *G. lozoyensis*

**Figure 6:**
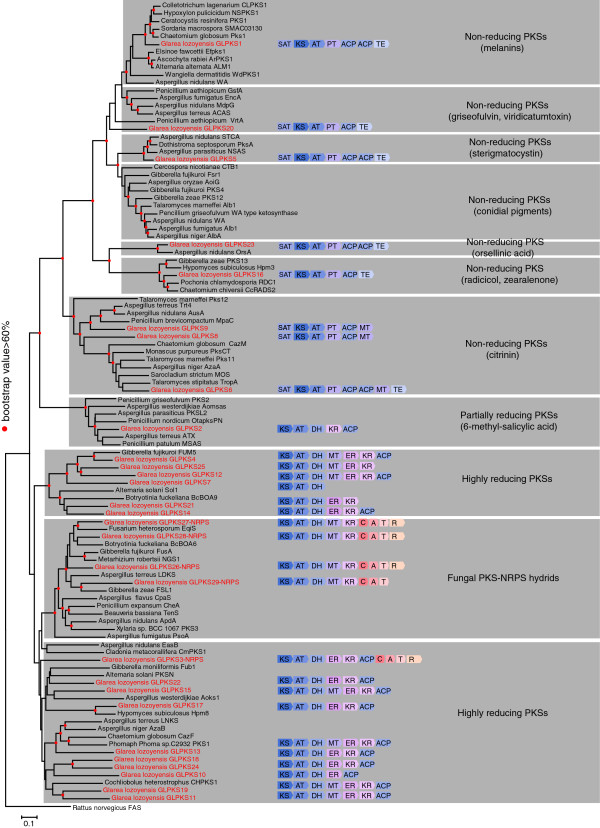
**Domain prediction and phylogenetic analysis of polyketide synthases (PKSs) and polyketide synthases-nonribosomal peptide synthetase hybrids (PKS-NRPS hybrids) in *G. lozoyensis *and other characterized fungal PKSs.** PKS and PKS-NRPS domains from *G. lozoyensis* were annotated by SMURF, anti-SMASH and SWISS-MODEL tools. SAT, starter unit acyltransferase domain; KS, ketosynthase domain; AT, acyltransferase domain; PT, product template domain; DH, dehydratase domain; ER, enoylreductase domain; KR, β-ketoacylreductase domain; MT, methyltransferase domain; ACP, acyl carrier protein; TE, thioesterase domain; A, adenylation domain; T, thiolation domain; C, condensation domain; R, reductive domain. Genealogy of PKSs and PKS-NRPSs was inferred by neighbor-joining analysis of the aligned amino acid sequences of the KS domains. Classification of PKSs and PKS-NRPSs sharing a common domain organization are highlighted by gray shading. Branch length indicates number of inferred amino acid changes. Red dots indicate branch nodes with >60% support. PKSs from *G. lozoyensis* are marked in red. See in Additional file 2: Table S3 for details of gene designations and their corresponding metabolites and references.

An unexpected feature of the *G. lozoyensis* genome was its remarkable diversity of polyketide biosynthetic pathways and having at least 29 recognizable core PKS genes (Figure 6). Domain structure analysis revealed eight non-reducing PKSs, one partially-reducing PKS, four PKS-NRPS hybrids encoding partially reducing polyketides [37] and 16 PKSs encoding for highly reducing polyketides, including GLPKS4 and one PKS-NRPS hybrid (GLPKS3-NRPS) (Figure 6). A phylogenetic tree based on amino acid sequences of the ketosynthase domains (KS) was constructed for the 24 PKSs and five PKS-NRPS hybrids in *G. lozoyensis* and 71 functionally characterized fungal PKSs encoding the products with known chemical structures (Figure 6, Additional file 2: Table S3). All four fungal-type PKS-NRPS hybrids (GLPKS26-NRPS, GLPKS27-NRPS, GLPKS28-NRPS, and GLPKS29-NRPS) were grouped with similar PKS-NRPS hybrids, such as those involved in the biosynthesis of the tetramic acids and HIV-1 integrase inhibitor equisetin (EqiS). Interestingly the four PKS-NRPS hybrids were also clustered with the HMG-CoA reductase inhibitor lovastatin (LDKS = LovB) which is proposed to be a truncated PKS-NRPS hybrid [38,39]*.* GLPKS8 and GLPKS9 were predicted to be non-reducing PKSs related to the PKSs responsible for biosynthesis of the metabolites mycophenolic acid and citrinin. GLPKS13 and three other *G. lozoyensis* PKSs (GLPKS10, GLPKS18, and GLPKS24) were grouped with the PKSs of lovastatin side chain (LNKS = LovF) [40] and the tetraketide acyl side chain of zaragozic acid A [41]. GLPKS19 and GLPKS11 shared significant homology with the T-toxin encoding gene CHPKS1 of *Cochliobolus heterostrophus*[42,43]. Six more *G. lozoyensis* PKSs (GLPKS4, GLPKS25, GLPKS12, GLPKS7, GLPKS14, and GLPKS21) clustered with the hepato- and nephro-toxic fumonisin B_1_ produced by *Gibberella fujikuroi*[44] and the solanapyrone Sol1 PKS of *Alternaria solani*[45]. The previously characterized GLPKS2, encoding for the biosynthesis of 6-methylsalicylic acid [20], grouped tightly with two other fungal 6-methylsalicylic acid PKSs, ATATX from *A. terreus* and MSAS from *Penicillium patulum*[46,47]. GLPKS1 has been previously identified as the *G. lozoyensis* melanin biosynthesis gene [21], and it clustered with other fungal di- and tetra-hydroxynaphthalene melanin biosynthesis genes, e.g. *Hypoxylon pulicicidum* (formerly *Nodulisporium* sp.) (NSPKS1) [48] and *Colletotrichum lagenarium* (CLPKS1) [49]. The ketosynthase sequence of *G. lozoyensis* GLPKS20 exhibited sequence similarities to genes involved in the biosynthesis of viridicatumtoxin [50]. Adjacent to the large groups of melanin and conidial pigment genes were the mycotoxin sterigmatocystin PKS (ANST) from *A. nidulans*[51] and the GLPKS5 from *G. lozoyensis*. Distantly related to the pigment PKSs was the *A. nidulans* orsellinic acid PKS protein OrsA [52,53], and GLPKS23 shared the same domain structure with OrsA. We speculated that orsellinic acid or related compounds may be produced by *G. lozoyensis*, and analysis of fermentations of *G. lozoyensis* confirmed that it produced isolecanoric acid (an orsellinic acid dimer) and pseudogyrophoric acid (a new orsellinic acid trimer) in certain culture media (Additional file 1: Figure S2). Therefore, we propose that GLPKS23 is responsible for orsellinic acid biosynthesis in *G*. *lozoyensis*. Cluster analysis revealed that a highly reducing PKS (GLPKS17) was proximal to a non-reducing PKS (GLPKS16) in the same cluster (Additional file 1: Figure S1). This tandem PKS structure was similar to that of the PKSs responsible for the biosynthesis of resorcylic acid lactones, e.g. radicicol and hypothemycin, and in fact, GLPKS16 appeared to be an ortholog of Hpm3 and RDC1 (Figure 6) [54,55].

**Figure 7:**
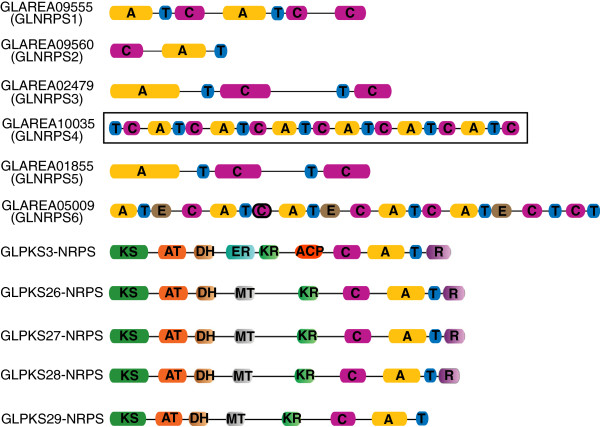
**Schematic representations of the functional domains in nonribosomal peptide synthetase (NRPS) and polyketide synthase-nonribosomal peptide synthetase hybrid (PKS-NRPS hybrid) proteins in *G. lozoyensis*.** A, adenylation domain; T, thiolation domain; C, condensation domain; E, epimerization domain; KS, ketosynthase domain; AT, acyltransferase domain; DH, dehydratase domain; ER, enoylreductase domain; KR, β-ketoacylreductase domain; MT, methyltransferase domain; ACP, acyl carrier protein; R, reductive domain. The pneumocandin-encoding NRPS (GLAREA10035 GLNPRS4) with six A-T-C modules is outlined.

**Figure 8:**
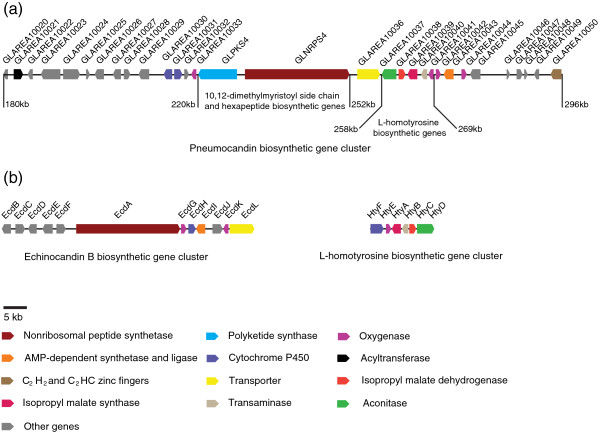
**Schematic representation of the pneumocandins and echinocandin B biosynthetic gene cluster.** (**a**) Pneumocandins biosynthetic gene cluster. (**b**) Echinocandin B biosynthetic gene clusters (including L-homotyrosine biosynthetic gene cluster) based on [8,24].

NRPSs include modules that incorporate amino acids into the final peptide product. Each module minimally contains three domains, the adenylation domain (A domain), the thiolation domain (T domain), and the condensation domain (C domain). In addition to its abundant and diverse PKS pathways, the *G. lozoyensis* genome harbored six NRPS genes. Three NRPSs (GLNRPS2, GLNRPS3, GLNRPS5), contained a single module, encoding products with a single amino acid, the other three NRPSs (GLNRPS1, GLNRPS4, GLNRPS6), were multi-modular, encoding products with more than one amino acids (Figure 7). Gene cluster analysis revealed that GLNRPS1 (with two modules), GLNRPS2 and one NRPS-like genes located in the same cluster flanked by three clavaminate synthases (oxygenases) and MFS general substrate transporter genes (Additional file 1: Figure S1). These data indicated that a hydroxylation tetrapeptide product may be formed and excreted. GLNRPS4, with six modules that encode a hexapeptide product and located in a cluster bordered by various modifying enzymes, was proposed to be responsible for pneumocandin biosynthesis (Figure 7 and Figure 8a). Domain analysis revealed that GLNRPS6 had five modules, and module 1, module 3, module 5 contain one epimerization (E) domain respectively. The *glnrps6* was located in a cluster flanked by one MFS general substrate transporter gene, and thus suggested that a pentapeptide with three D-amino acids may be formed and excreted. Thirteen additional NRPS-like genes clusters were identified in *G. lozoyensis*, and some of them were located in clusters flanked by cytochrome P450, methyltranferase and transporter genes, thus indicating some hydroxylation and methylation products may be formed and excreted (Additional file 1: Figure S1).

To detect the classes of terpene synthases (TSs) in *G. lozoyensis*, the homologous sequences were analyzed by using BLAST at NCBI (http://www.ncbi.nlm.nih.gov/) (Additional file 2: Table S1). The richness of TSs, compared to related genome-sequenced fungi [27], revealed a great potential for *G. lozoyensis* to produce terpenoids. Three TS genes (GLAREA03340, GLAREA04931, GLAREA10578) encoded geranylgeranyl pyrophosphate synthase and geranylgeranyl transferase, and indicated these genes may be responsible for diterpene and carotenoid biosynthesis [56]. Two genes (GLAREA04679, GLAREA02940) encoding farnesyl pyrophosphate synthetase and farnesyl transferase indicated that sesquiterpenes may be formed [56]. Among these TS genes, only three (GLAREA11903, GLAREA03340, GLAREA08044) were located in gene clusters (Additional file 1: Figure S1). Two DMATS genes were found in the *G. lozoyensis* genome, and one gene (GLAREA04251) was located in a cluster downstream of another core PKS gene GLPKS9, signifying that a polyketide linked with dimethylallyl tryptophan may be the cluster’s end product.

### Identification of GLNRPS4 involving in pneumocandin biosynthesis in *G. lozoyensis*

The lipohexapeptide pneumocandin consists of two key components: a six-amino acid cyclic peptide and a 10,12-dimethylmyristoyl polyketide side chain [57]. Even though no such products are currently known from functionally characterized PKS-PKS hydrids [38], it is reasonable to consider that pneumocandins might be encoded by one of the PKS-NRPS hybrid proteins. However, in echinocandin B, the lipid side chain was thought to be derived from the cytoplasmic fatty acid pool [24]. Furthermore, domain analysis precluded the five PKS-NRPS hybrid proteins from pneumocandin biosynthesis because the hybrids contained only one A-T-C module, which could only incorporate one amino acid residue in the polyketide chain (Figure 7). Domain analysis of the six NRPS proteins showed that locus GLAREA10035 contained a NRPS with six A-T-C modules (designated as *glnrps4* and boxed) (Figure 7). Therefore, locus GLAREA10035 was the only plausible candidate. GLNRPS4, inferred to be responsible for the biosynthesis of the cyclic-hexapeptide core of the pneumocandins, comprised 7,192 amino acids and was encoded by a gene with two introns (Additional file 1: Figure S3). GLNRPS4 encompassed 20 domains grouped into six modules each corresponding to one of the six amino acid incorporated monomers (Figure 7). The first module of GLNRPS4 had a unique T-C-A-T-C domain structure that differed from the other five modules which contained A-T-C domain structures. Two bioinformatics programs were used for substrate prediction, and both predicted that the third module encoded for proline [58,59]. However, neither program consistently predicted substrate specificities for the other five modules.

### Analysis of the PKS-NRPS gene cluster for pneumocandin biosynthesis

Gene analysis of 50 kb of DNA flanking GLNRPS4 revealed a typical gene cluster for fungal secondary metabolite biosynthesis (Figure 8a). Immediately upstream of GLNRPS4 was the *glpks4* gene which encodes a PKS of 2,531 amino acids with eight introns (Additional file 1: Figure S3). Moreover, the PKS encoded by *glpks4* contained a methyltransferase domain that would be required for the biosynthesis of methyl group-containing fungal polyketides; the pneumocandin polyketide side chain contains two methyl groups (Figure 1a, Additional file 1: Figure S3) [5,57]. In addition to GLNRPS4 and GLPKS4, two other genes in this cluster stood out, GLAREA10021 encoding an acyltransferase and GLAREA10043 encoding an acyl-CoA ligase (Figure 8a). Labeling experiments at Merck revealed that GLPKS4 assembled a myristate from an acetyl starter, whereas methionines provided two methyl groups to form the 10,12-dimethylmyristoyl side chain [18]. Although functional characterizations will be necessary to define how each gene contributes to the biosynthetic mechanism, based on the above analyses and those of the echinocandin B and emericellamide pathways [24,60], a hypothetical model of the pneumocandin biosynthetic pathway can be formulated from the four genes, GLNRPS4, GLPKS4, acyltransferase (GLAREA10021), and acyl-CoA ligase (GLAREA10043). The model predicts that 10,12-dimethylmyristoyl side chain is released from GLPKS4 as a carboxylic acid that is converted to a CoA thioester by the acyl-CoA ligase (GLAREA10043), and then loaded onto the acyltransferase (GLAREA10021). The polyketide intermediate could then be shuttled to the first thiolation (T) domain of GLNRPS4, followed by its acylation to 4,5-dihydroxyorinithine to trigger elongation of the cyclic hexapeptide. Like other fungal NRPS and PKS gene clusters, the *glpks4* and *glnrps4* are positioned within a cluster that contains genes encoding for one or more cytochrome P450s, clavaminate synthase-like proteins (oxygenases), zinc finger transcription factors, and an ABC transporter (Figure 8a). It has been demonstrated that proline 3-hydroxylase and proline 4-hydroxylase, which are members of the 2-oxoglutarate-dependent dioxygenase class, can convert proline to 3-hydroxyproline and 4-hydroxyproline [61]. Two of the four oxygenases (GLAREA10033, GLAREA10041, GLAREA10042, and GLAREA10044) in the gene cluster were presumed to be involved in proline conversion. Two cytochrome P450 monooxygenases (GLAREA10030 and GLAREA10031) were classified in the CYP 512A family by the P450 database (http://www.cyped.uni-stuttgart.de/) which might be responsible for the hydroxylation of the amino acids. These oxygenases were also presumably involved in an oxidative mechanism for the conversion of leucine to methyl proline [19]. The putative zinc finger transcription regulator (GLAREA10050) belongs to the C_2_H_2_ and C_2_HC zinc finger superfamily which are DNA-binding proteins and transcription factors [62]. Some members of this family are pathway-specific transcription regulators of secondary metabolite biosynthesis, e.g., Rua1 that activates the ustilagic acid biosynthesis gene cluster in *Ustilago maydis*[63]. Therefore, the zinc finger protein GLAREA10050 most likely regulates transcription of the *glpks4* and *glnrps4* genes. ABC transporters are ubiquitous membrane proteins with the ability to pump a variety of substrate specificities of endogenous and exogenous toxic compounds [64,65]. The ABC transporter (GLAREA10036) in the cluster possibly secretes antifungal pneumocandins, thus avoiding of intracellular accumulation and ameliorating the toxicity to the producing cells.

Finally, a putative biosynthetic pathway for L-homotyrosine, the non-proteinogenic amino acid in the pneumocandin peptide core’s fourth position, sits downstream of GLNPRS4 (Figure 8a)*.* This set of five contiguous genes showed significant identity to the L-homotyrosine pathway of *E. rugulosa*[24] (Figure 8b), although the direction of transcription was inverted in two of the five genes, and consisted of GLAREA10037, an aconitase (62% identity to *hyt*D), GLAREA 10038, an isopropyl malate dehydrogenase (71% identity to *hty*C), GLAREA10039, a 2-isopropyl malate synthase (63.7% identity to *hyt*A), and GLAREA10040, an aminotransferase (64% identity to *hyt*B), and GLAREA10041, a non-heme dioyxgenase (63.8% identity to *hyt*E). However, unlike the L-homotyrosine pathway of *E. rugulosa*, the cytochrome P450 oxygenase gene corresponding to *hyt*F, was absent (Figure 8b).

### Functional analysis of *glpks4* and *glnrps4* in pneumocandin biosynthesis

**Figure 9:**
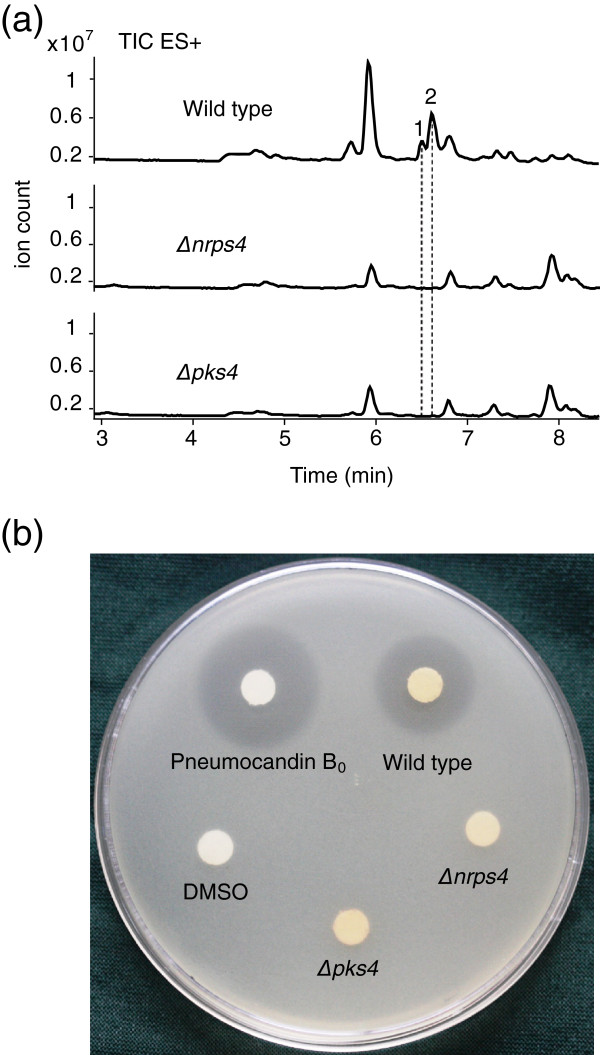
**Chemical and functional analysis of pneumocandins produced by wild-type and mutant strains of *G. lozoyensis*.** (**a**) HPLC-MS profiles of chemical extracts from the wild-type (WT), *glnrps4* deletion mutant, and *glpks4* deletion mutant of *G. lozoyensis.* Full-scan + mode spectra were acquired in over a scan range of *m/z* 80–1,200. When grown in FGY broth, pneumocandin B_0_ (peak 1 *m/z* = 1065) and pneumocandin A_0_ (peak 2 *m/z* = 1079) were detected in the WT strain. Deletion of *glnrps4* and *glpks4* abolished pneumocandin B_0_ and pneumocandin A_0_ production in the mutant strains. (**b**) Antifungal activity of culture extracts from the WT and corresponding inactive extracts from *glnrps4* and *glpks4* mutants of *G. lozoyensis.* Purified pneumocandin B_0_ (5 mg mL^-1^) and DMSO (100%) were used as positive and negative controls, respectively.

To verify whether the gene cluster was responsible for pneumocandin biosynthesis, *glnrps4* and *glpks4* were knocked out by homologous replacement with an *Agrobacterium*-mediated transformation protocol developed previously for *G. lozoyensis*, and the deletions were verified by PCR analysis (Additional file 1: Figure S4b and S4c). Twelve and ten positive transformants were recovered for the GLNRPS4 and GLPKS4 knockouts, respectively. After growing the fungi in FGY medium and comparative analysis of the extracts by HPLC-MS using purified pneumocandin B_0_ as a standard, the two major pneumocandins (A_0_ and B_0_) were produced by the *G. lozoyensis* WT strain as expected, but the pneumocandins were absent in the *glnrps4* and *glpks4* knockout mutants (Figure 9a). Consistent with earlier observations [5], the WT strain produced pneumocandin A_0_ in larger quantities than pneumocandin B_0_ (Figure 9a, Additional file 1: Figure S4d). Antifungal assays showed that crude extracts from the WT strain caused zones of inhibition against the yeast *C. albicans*, whereas the crude extracts from mutants *Δglnrps4* and *Δglpks4* were inactive (Figure 9b). These results demonstrated that both *glnrps4* and *glpks4* were essential for biosynthesis of the pneumocandin core structure as predicted.

## Discussion

Sequenced genomes are yielding substantial evidence for a richness of secondary metabolite pathways among the major kinds of fungi, well beyond that imagined to date, and the number of sequenced genomes is growing exponentially [66,67]. With the advance of next-generation sequencing technology, genome sequencing is evolving as an essential tool to decipher novel genes and gene clusters involved in biosynthesis of different metabolites in fungi [22,68]. For example, the biosynthetic pathway of the insecticidal cyclodepsipeptide destruxins was recently elucidated in the insect fungal pathogen *Metarhizium robertsii* by genomic sequencing [69]. Genomic mining of several *Aspergillus* spp. has led to the elucidation of biosynthetic pathways of multiple bioactive compounds, including terrequinone A [70], emericellamide [60], aspyridones [71],pyripyropene A [72] and echinocandin B [24].

Genomic sequence analysis showed that *G. lozoyensis* has the potential to produce a diverse array of natural products. The genome was predicted to encode 49 gene clusters that contribute to its secondary metabolome, significantly higher than that of *A. sarcoides*, also of the Helotiaceae [28], and in the same order of magnitude as that of *B. cinerea*, *S. sclerotiorum*, and other sequenced Leotiomycetes [27,73]. Most of the ketosynthase domains of the 24 PKSs and five PKS-NRPS hybrids could be clustered with PKSs that were responsible for the biosynthesis of bioactive polyketides and polyketide-nonribosomal peptide hybrids (Figure 6). However, biosynthetic functions for only two of the 49 secondary metabolite-encoding genes in *G. lozoyensis* were previously validated (GLPKS1 for melanin and GLPKS2 for 6-methylsalicylic acid) [20,21]. Many secondary metabolites are fusions of nonribosomal peptides and polyketides, in which a PKSs and NRPSs interface and contribute to the same pathway end product [38,60,74]. Because the NRPS portion in each of the five PKS-NRPS hybrids in *G. lozoyensis* genome contains only one A-T-C module, one amino acid is predicted to be added to the polyketide produced by the PKS portion of the cluster, similar to ApdA in *A. nidulans* and ATEG00325 in *A. terreus*, which are involved in the biosynthesis of aspyridones and flavipucine, respectively [71,75].

Comparing the rich genetic potential for secondary metabolites in the *G. lozoyensis* genome, only pneumocandins were previously identified from the fungus. In an attempt to find additional chemistries, we identified isolecanoric acid and pseudogyrophoric acid as two new fermentation products of *G. lozoyensis* (Additional file 1: Figure S2). Therefore, majority of the secondary metabolites in *G. lozoyensis* remain to be characterized. Despite the advances in the field of microbial secondary metabolite biosynthesis, how the basic biology, ecology, and trophic strategies of microorganisms relate to their secondary metabolite production remains poorly understood. Application of efficient strategies to mine the metabolite-encoding gene clusters in *G. lozoyensis* and other poorly known fungi, while identifying their corresponding metabolites, presents a challenge and opportunity for natural products discovery.

GLNRPS4 and GLPKS4 are centrally located in the pneumocandin biosynthetic gene cluster, and how they cooperate with other genes in the cluster is still speculative. Even though they are independently transcribed and translated, their transcription is likely to be synchronized or co-regulated. The first module in GLNRPS4 has a unique T-C-A-T-C structure, and the first T domain in the T-C-A-T-C module is suggest to accept thiolated intermediates as found in emericellamide biosynthesis [60] or adenylated substrates similar to yersiniabactin biosynthesis [76]. Thus, the first T domain in the T-C-A-T-C module of GLNRPS4 could be responsible for accepting the incoming 10,12-dimethylmyristoyl side chain intermediate, whereas the second T domain would accept the 4,5-dihydroxyornithine adenylated by the module’s A domain. Threonine, 4-hydroxyproline, 4,5-dihydroxyhomotyrosine, 3-hydroxyglutamine and 3-hydroxyproline/3-hydroxy-4-methylproline would be sequentially added to the growing chain consistent with the *in silico* prediction that the A3 in GLNRPS4 is specific to proline [8,58,59]. Like many other NRPSs [60,77,78], the carboxyl terminal of GLNRPS4 lacks a thioesterase (TE) domain, suggesting that a dedicated TE is not required for pneumocandin cyclization. The last C domain of GLNRPS4 is proposed to be responsible for cyclization by condensation to form the peptide bond between 4,5-dihydroxyornithine and 3-hydroxyproline/3-hydroxy-4-methylproline. This proposal is consistent with the fact that the C domain has a HAEYD motif similar to the active site signature in the terminal C domain of cyclosporine synthetase (HSLYD) which is responsible for cyclization of cyclosporine in *Tolypocladium inflatum* and siderophore synthase SidC (HSLYD) involved in cyclization of the siderophore ferricrocin in *A. nidulans*[79,80]. The proposed biosynthetic sequence also parallels that proposed for echinocandin B [8,24]. Five of the six amino acids in the cyclic hexapeptide were hydroxylated, and hydroxylations of the two proline residues in pneumocandin B_0_ were catalyzed by a proline-3-hydoxylase and a proline-4-hydoxylase [61]. The enzyme responsible for hydroxylation of 4-methylproline derived from leucine in pneumocandin A_0_ may also be a proline 3-hydroxylase as 4-methylproline is an analogue of l-proline [19].

Other genes downstream of the GLNRPS4 that are likely involved the biosynthesis are the putative acyl-CoA ligase GLAREA10043 which shares 43% identity with EasD which converts polyketide carboxylic acid to a CoA thioester in emericellamide biosynthesis in *A. nidulans*[60]. The putative acyltransferase GLAREA10021 in the cluster shares more than 65% identity with the cholesterol acyltransferases from *Cordyceps militaris*[81]. Existence of these two genes suggests that the polyketide intermediate was first synthesized by GLPKS4, and then shuttled to the first T domain of GLNRPS4 mediated by the two enzymes, in a fashion similar to the emericellamide biosynthetic pathway [60]. Surprisingly and unlike the echinocandin B pathway [24], the putative pathway for the homotyrosine residue of the pneumocandin peptide core also sits downstream, and presumably L-homotyrosine biosynthesis is synchronized with the rest of the pathway.

The pneumocandin and echinocandin B pathways have some striking commonalities, yet obviously differ in their organization. The most obvious similarity is the high degree of identity between *ecdA* and *glnrps4* (60.8% identity over 22.7 kb, 55.2% identity over 7218 aa), and both have the same orientation in transcription and functional modules (TCATCATCATCATCATCATCT). Likewise, the genes of the L-homotyrosine pathway are highly similar, although their physical proximities to the core NRPS differ. Both pathways also contain a number of oxygenases that, in the case of echinocandin B, tailor the multiple hydroxyl or diol groups of the amino acid core, but once again their physical location and order are significantly rearranged. However, the inclusion a PKS for side chain biosynthesis and its proximity for immediate loading onto the first thiolation domain, along with close proximity of the L-homotyrosine gene cluster and a possible zinc finger regulatory protein would likely confer greater metabolic autonomy to the pneumocandin pathway. The remarkable similarity between the echinocandin and pneumocandin pathways and especially the high degree of sequence homology between the amp-binding domains of GLNRPS4 and EcdA raises questions about pathway acquisition through horizontal gene transfer among fungi [82,83]. However, with only two echinocandin type pathways characterized thus far, speculation on why fungi from evolutionary lineages, Eurotiomycete (*E. rugulosa*) versus Leotiomycete (*G. lozoyensis*) that diverged 100s of millions of years ago, would share or converge on such similar molecular scaffolds is still premature. Elucidation of additional echinocandin type pathways in the Eurotiomycete, e.g., aculeacin and mulundocandin, and in the Leotiomycetes, e.g. FR901379 (WF11899A) and cryptocandin would yield evidence to determine a possible echinocandin progenitor and the probable directionality in gene recruitment or losses during the evolution of the echinocandin-pneumocandin gene clusters, as well as the significance of these potent cell wall-modifying metabolites to the fungi that produce them.

Elucidation of the pneumocandin biosynthetic pathway in *G. lozoyensis* paves the way for designing experimental procedures to enhance the production titer of the pneumocandins or engineering analogues with improved oral availability or broader spectrum of antifungal activities. Deletion of other PKS and NRPS genes could potentially reduce metabolic competition for substrates to GLPKS4 and GLNRPS4 and therefore increase the titers of pneumocandin B_0_, in a manner similar to the disruption of GLPKS1 melanin gene in *G. lozoyensis* which doubled pneumocandin production titer [21]. Elimination, inactivation, addition or modification of the specificity of domains to GLPKS4 and GLNRPS4 could result in new pneumocandin derivatives via biocombinatorial chemistry approaches for the discovery and development of improved antifungal therapy.

## Conclusion

The *Glarea lozoyensis* genome was sequenced, completely assembled and thoroughly annotated. The menu of secondary metabolites encoding genes was predicted from the genome, thus providing a greater understanding the complexity of primary and secondary metabolism in fungi from the yet poorly studied Leotiomycetes. The biosynthetic gene cluster responsible for pneumocandin was predicted *in silico* and identified by core gene *glpks4* and *glnrps4* knockouts and bioassay experiments. The data from this study will form the basis for a more detailed functional analysis of pneumocandin biosynthetic pathways and enable the identification of other antifungal lipohexapeptide pathways in other fungi, of which both will be essential for increasing pneumocandin production and for generating new pneumocandin and echinocandin derivatives via biocombinatorial chemistry approaches.

## Methods

### Fungal and bacterial strains, vectors, and other reagents

The original pneumocandin producing strain of *G. lozoyensis* ATCC 20868 was obtained from American Type Culture Collection (ATCC) and was used as the wild-type recipient in *Agrobacterium*-mediated transformation experiments. The *Escherichia coli* strain DH5α was used in plasmid manipulations. *Agrobacterium tumefaciens* AGL-1 was described by Lazo *et al.*[84]. Plasmid pAg1-H3 was described by Zhang *et al*. [21], pEASY-T3 vector was from TransGen Biotech (Beijing, China), and pMD18-T vector was from Takara Biotech (Dalian, China). The pneumocandin B_0_ standard was from Molcan Corporation (Ontario, Canada). LYCP-5 medium, FGY medium and conditions for *G. lozoyensis* fermentation were described by Connors *et al*. [85]. M-100 and IMAS mediums were described by Wang *et al*. [69]. Potato dextrose agar (PDA) and Sabouraud dextrose agar (SDA) were from Becton Dickinson (Franklin Lakes, New Jersey, USA). *E. coli* and *A. tumefaciens* AGL-1 were cultured as described by Zhang *et al*. [21]. Restriction endonucleases and DNA modifying enzymes were from New England Biolabs (Beverly, Massachusetts, USA).

### DNA isolation and sequencing

Genomic DNA of *G*. *lozoyensis* ATCC 20868 was extracted as previously described by Zhang *et al*. [21]. Genomic DNA libraries with 500–800 bp inserts were constructed and sequenced with a Roche 454 GS FLX at the Chinese National Human Genome Center in Shanghai. A library with 3 kb inserts was constructed and sequenced with Illumina Genome Analyzer using the protocols as described for genomic sequencing of *Cordyceps militaris*[81]. The genome sequences were assembled using Newbler software (ver. 2.3) and SSPACE (http://www.baseclear.com/dna-sequencing/data-analysis/bioinformatics-tools/).

### *G. lozoyensis* genome annotation, orthology and phylogenomic analyses

The *G. lozoyensis* genome was annotated with Augustus (http://bioinf.uni-greifswald.de/augustus) by referencing annotated genome of *Botrytis cinerea*. GeneID and GeneMark-ES were additionally used for open reading frames prediction in *G. lozoyensis*[86,87]*.* Repetitive sequences in the genome were identified by BLAST against the RepeatMasker library [88] and by *de novo* repetitive sequence search using RepeatModeler (http://www.repeatmasker.org/RepeatModeler.html). Transfer RNAs (tRNAs) were identified with tRNAscan-SE [89]. Ribosomal RNAs (rRNAs) were predicted by a BLAST search with known rRNA modules from other fungal genomes. Whole genome protein families were classified by InterproScan analysis (http://www.ebi.ac.uk/interpro/) and BLAST against Kyoto Encyclopedia of Genes and Genomes (KEGG) database using KEGG Automatic Annotation Server (KAAS: http://www.genome.jp/kegg/kaas/). Carbohydrate-active enzymes from *G. lozoyensis* and reference fungi (Additional file 2: Table S2) were classified by local Blastp searching against a library of catalytic and carbohydrate-binding module enzymes [90]. PKS, NRPS, DMATS and related gene clusters were predicted by programs SMURF and anti-SMASH [68] and by manual annotation.

A total of 878 common orthologous genes were identified using the InParanoid pipeline in the selected fungal genomes (Additional file 2: Table S2) [91], and aligned with Clustal W (ver. 2.0). A maximum likelihood phylogenomic tree was created using the concatenated amino acid sequences in PAUP* 4.0 (beta 10 Win) with heuristic searches [92]. Characters were treated as unordered and gaps were regarded as missing data. Bootstrap support for internal branches was estimated by analysis of 1,000 pseudo replicates. Reference fungi used to construct the phylogenetic tree were described elsewhere (Additional file 2: Table S2 and ref. [26]). All internal transcribed spacer (ITS) sequences were aligned with Clustal W (ver. 2.0), and a neighbor-joining phylogenetic tree was generated with the program PAUP* 4.0 (beta 10 Win) using 1,000 bootstrap replicates and a Jukes-Cantor substitution model with pairwise deletion for gaps or missing data [92].

### Phylogenetic analysis of PKS and PKS-NRPS genes

KS domains from fungi with PKS genes proven to be responsible for metabolites biosynthesis (Additional file 2: Table S3), PKSs and PKS-NRPS hybrids in *G. lozoyensis* (Additional file 2: Table S4) were identified by the program anti-SMASH [93] or visually in multiple alignments. All KS domains from PKS were aligned with Clustal X (ver. 2.0), and analyzed phylogenetically with MEGA 5.0 using a Jones-Taylor-Thornton substitution model, a pair-wise deletion for gaps or missing data, and a 1,000 bootstrap replications test [94]. The tree was rooted with the KS domain of the rat fatty acid synthase (Figure 6).

### Gene knockout of *glnrps4* and *glpks4*

To verify function of the predicted pneumocandin gene cluster, *glnrps4* and *glpks4* deletion were conducted using method reported by Zhang *et al*. [21]. To verify function of the predicted pneumocandin gene cluster, gene knockout constructs for *glnrps4* and *glpks4* were created. Briefly, the flanking regions of the target genes were amplified using different primer pairs (Additional file 2: Table S5) and ligated into the binary vector of pAg1-H3 containing the hygromycin resistance gene to form pAg1-H3-nrps4 and pAg1-H3-pks4. The constructs were introduced into *G. lozoyensis* by *Agrobacterium*-mediated transformation using method reported by Zhang *et al*. [21] with slight modification. Conidia for transformation were harvested into sterile 0.05% Tween-20 followed with 2 times of wash with distilled water and then suspended into 0.5-1.0 mL sterile water (10^6^ spores mL^-1^). One hundred microliters of *G. lozoyensis* and 100 μL of *A. tumefaciens* (OD_660 nm_ = 0.6-0.8) were mixed and spread on the IMAS agar plate and co-incubated at 28°C for 2 d. The co-culture of *A.tumefaciens* and *G. lozoyensis* was covered with M-100 supplemented with 300 μg mL^-1^ cefotaxime and 100 μg mL^-1^ hygromycin B, and incubated at 25°C for 2–3 weeks before isolating hygB resistant colonies. The transformants were purified by single conidium isolation and the gene knockout transformants were verified by PCR using multiple primers (Additional file 2: Table S5).

### HPLC-MS analysis of pneumocandins

Fermentation and pneumocandin extraction protocols were described by Petersen *et al*. [95]. HPLC separation was performed on an Agilent Zorbax Extend-C18 1.8 μm 2.1 × 50 mm column using an Agilent 1200 Series system (Agilent, USA). The total flow rate was 0.3 mL min^-1^; mobile phase A was with 0.1% formic acid and mobile phase B was acetonitrile. The total elution program was 25 min. Gradient elution began with 30% B for 0.5 min, changed to 70% B over 3.5 min, changed to 100% B over 8 min, maintained at 100% B for 5 min, to 30% B over 0.5 min, and re-stabilized for 7.5 min prior the next injection. The column temperature was maintained at 40°C. The injection volume was 10 μL.

Mass spectra were acquired with an Agilent Accurate-Mass Quadrupole–Time-of-Flight mass spectrometry (Q-TOF/MS) 6520 system in the positive ionization mode. For Q-TOF/MS conditions, fragmentor and capillary voltages were kept at 130 and 3,500 V, respectively. Nitrogen was supplied as the nebulizing and drying gas. Temperature of the drying gas was set at 30°C. The flow rate of the drying gas and the pressure of the nebulizer were 10 L min^-1^ and 25 psi, respectively. Full-scan spectra were acquired over a scan range of *m/z* 80–1,200 at 1.03 spectra s^-1^.

### *Candida albicans* zone of inhibition (ZOI) assays

Antifungal activity of the WT, *glnrps4* and *glpks4* gene deletion mutants of *G. lozoyensis* was measured by a zone of inhibition assay against the human fungal pathogen *Candida albicans* SC 5314. Ten-mL liquid culture of the wild-type or *glnrps4* and *glpks4* gene deletion mutants of *G. lozoyensis* were lyophilized in a vacuum freeze dryer, and 10 mL methanol were added and thoroughly mixed. After 1 h of orbital shaking, the mixtures were first centrifuged at low speed, the supernatant was transferred to glass tubes, and then DMSO (2 mL) was added to solubilize any metabolites precipitated during evaporation. The samples were concentrated to 2 mL under a warm N_2_ stream during orbital shaking. The final samples were 5× whole broth equivalents including 100% DMSO relative to original culture volume.

*Candida albicans* SC 5314 cells grown on SDA plates were inoculated into 10 mL of Sabouraud dextrose broth and incubated overnight at 30°C. The *C. albicans* suspension was adjusted to an optical density of 0.4 at 660 nm and added to SDA in the proportion of 30 mL L^-1^. Twenty-mL aliquots of the seeded agar media were poured into 9-cm Petri plates. Pneumocandin B_0_ (5 mg mL^-1^) and 100% DMSO were used as positive and negative controls. The extracts prepared from liquid culture of *G. lozoyensis* and the controls (10 μL) were applied to paper discs on the surface of the seeded assay plates. The plates were incubated at 30°C for approximately 20 h and ZOIs were measured and photographed.

### Production, purification and identification of isolecanoric and pseudogyrophoric acids

Isolecanoric acid and the new compound pseudogyrophoric acid were isolated from the extract of *G. lozoyensis* ATCC 20868 grown in MV8 medium (V8 juice 200 mL, maltose 75 g, soy flour 1 g, L-proline 3 g, MES 16.2 g, distilled H_2_O 800 mL) at 22°C on a rotary shaker at 220 rpm for 14 d. The isolation procedure, mass spectra are summarized in Additional file 1: Figure S2.

## Abbreviations

WT: Wild-type; NRPS: Non-ribosomal peptide synthetase; PKS: Polyketide synthase; Mb: Megabase; CAZymes: Carbohydrate active enzymes; GH: Glycoside hydrolases; GT: Glycosyl transferases; PL: Polysaccharide lyases; CE: Carbohydrate esterases; CBM: Carbohydrate binding modules; TS: Terpene synthase; DMATS: Dimethylallyl tryptophane synthase; PKS-NRPS hybrids: Polyketide synthase-nonribosomal peptide synthetase hybrids; KS: Ketosynthase domains; HR-PKS: Highly reduced PKS; NR-PKS: Non reduced PKS; A: Adenylation; T: Thiolation; C: Condensation; TE: Thioesterase; ITS: Internal transcribed spacer; ZOI: Zone of inhibition.

## Competing interests

The authors declare that they have no competing interests.

## Authors’ contributions

ZA and XL designed research; LC, QY, F O-L and MX performed research; LC, XZ, CW, SL, YC and GB analyzed data; and LC, QY, GB, ZA, and XL wrote the paper. All authors read and approved the final manuscript.

## Supplementary Material

Additional file 1 **Figures that provide support information for the main text. Figure S1.** provides the 49 secondary metabolite biosynthetic gene clusters in the *G. lozoyensis* genome. **Figure S2** lists materials and methods for purification and characterization of additional metabolites from *G. lozoyensis* ATCC 20868 grown on MV8 medium. **Figure S3** shows gene structure of GLNRPS4 and GLPKS4. **Figure S4** summarizes strategy for the construction of GLPKS4 and GLNRPS4 gene deletion mutants. Click here for file

Additional file 2 Tables that provide support information for the main text. **Table S1** shows the homologous analysis of terpene gene in *Glarea lozoyensis*. **Table S2** lists the fungal genomes used for phylogenomic analyses and CAZymes analysis. **Table S3** provides supporting data for Figure 6 and the list of 71 functionally characterized fungal PKSs and PKS-NRPSs hydrids, their gene designations, the principal pathway end product, and references. **Table S4** shows polyketide synthases (PKSs) in the *G. lozoyensis* genome used for phylogenetic tree construction. **Table S5** summarizes primers used for genes deletion and mutants verification. Click here for file

## Accession number

The Whole Genome Shotgun project has been deposited at DDBJ/EMBL/GenBank under the accession ALVE00000000 *Glarea lozoyensis* ATCC 20868.
