# Supplementary material for: Genomics-driven discovery of the pneumocandin biosynthetic gene cluster in the fungus Glarea lozoyensis
Source: BMC Genomics. 2013 May 20;14:339. doi: 10.1186/1471-2164-14-339 (PMC3672099; doi:10.1186/1471-2164-14-339)
Supplement: Additional file 1 — Figures that provide support information for the main text. Figure S1. provides the 49 secondary metabolite biosynthetic gene clusters in the G. lozoyensis genome. Figure S2 lists materials and methods for purification and characterization of additional metabolites from G. lozoyensis ATCC 20868 grown on MV8 medium. Figure S3 shows gene structure of GLNRPS4 and GLPKS4. Figure S4 summarizes strategy for the construction of GLPKS4 and GLNRPS4 gene deletion mutants. [file 1471-2164-14-339-S1.pdf]

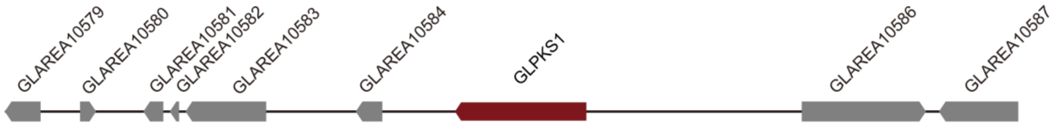

Hypothetical protein (GLAREA10579, GLAREA10580, GLAREA10582, GLAREA10587) Zn2/Cys6 DNA-binding (GLAREA10583)

Short-chain dehydrogenase/reductase SDR (GLAREA10581) Fungi TrN (GLAREA10584) Ankyrin repeat (GLAREA10686)

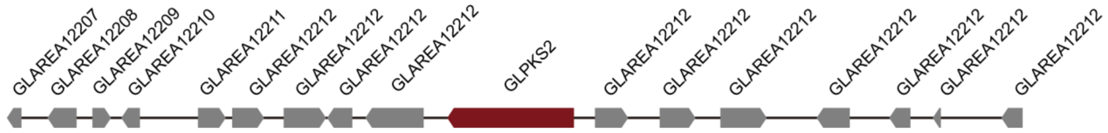

Hypothetical protein (GLAREA12210, GLAREA12212, GLAREA12213, GLAREA12215, GLAREA12220, GLAREA12221, GLAREA12222, GLAREA12223)

Short-chain dehydrogenase/reductase SDR (GLAREA12207, GLAREA12211) Radical SAM enzymes (GLAREA12208) C-8 sterol isomerase (GLAREA12209)

Alpha/beta-Hydrolases (GLAREA12214) FAD/NAD(P)-binding (GLAREA12217) Di-copper centre-containing (GLAREA12218)

MFS general substrate transporter (GLAREA12219)

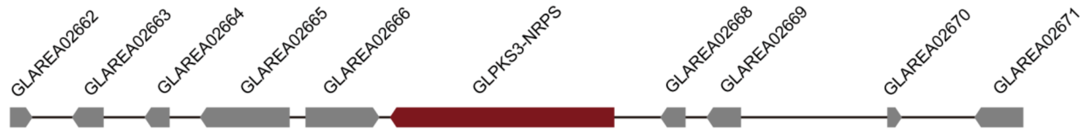

Hypothetical protein (GLAREA02662, GLAREA02668, GLAREA02670) Metallo-dependent phosphatases (GLAREA02663)

T-snare proteins (GLAREA02664) Ankyrin repeat (GLAREA02665) Magnesium transport protein CorA (GLAREA02666)

MFS general substrate transporter (GLAREA02669) Zn2/Cys6 DNA-binding (GLAREA02671)

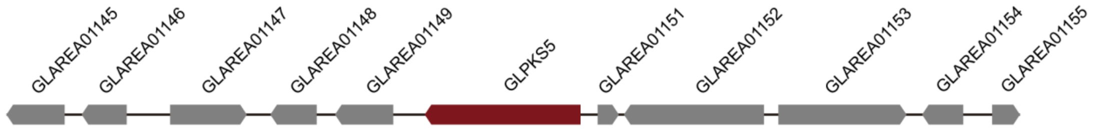

Hypothetical protein (GLAREA01145, GLAREA01146, GLAREA01148) Serine/threonine protein kinase (GLAREA01147)

Hlycosidases (GLAREA01149) Short-chain dehydrogenase/reductase SDR (GLAREA01151) Zn2/Cys6 DNA-binding (GLAREA01155)

FabD/lysophospholipase-like (GLAREA01152) Thiolase-like (GLAREA01153) MFS general substrate transporter (GLAREA01154)

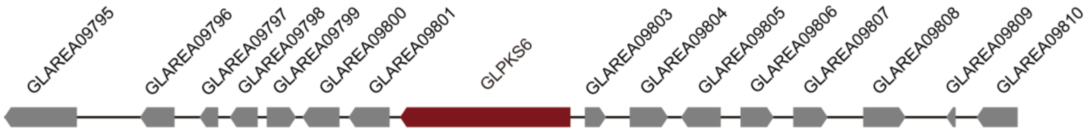

Calcium ATPase (GLAREA09795) Hypothetical protein (GLAREA09796, GLAREA09809, GLAREA09798)

FAD/NAD(P)-binding (GLAREA09799, GLAREA09800, GLAREA09804, GLAREA09807) GroES-like (GLAREA09803)

Zn2/Cys6 DNA-binding (GLAREA09801, GLAREA09808) MFS general substrate transporter (GLAREA09805, GLAREA09806)

Mannose-binding lectins (GLAREA09810) Alpha/beta-Hydrolases (GLAREA09797)

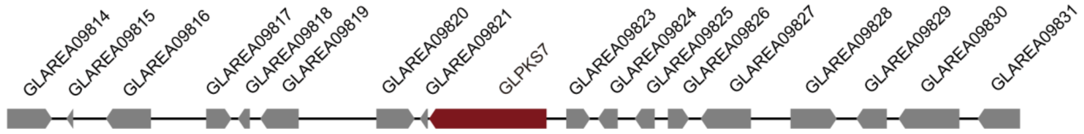

Hypothetical protein (GLAREA09814, GLAREA09815, GLAREA09816, GLAREA09818, GLAREA09828, GLAREA09829)

Bacterial luciferase-like (GLAREA09817) Cytochrome P450 (GLAREA09819, GLAREA09820) ACP-like (GLAREA09821)

Alpha/beta-Hydrolases (GLAREA09823) Ribonuclease Rh-like (GLAREA09824) Class I glutamine amidotransferase-like (GLAREA09825)

Glycerol-3-phosphate (1)-acyltransferase (GLAREA09826) P-loop containing nucleoside triphosphate (GLAREA09827)

Zn2/Cys6 DNA-binding (GLAREA09830) Sodium/phosphate symporter, putative (GLAREA09831)

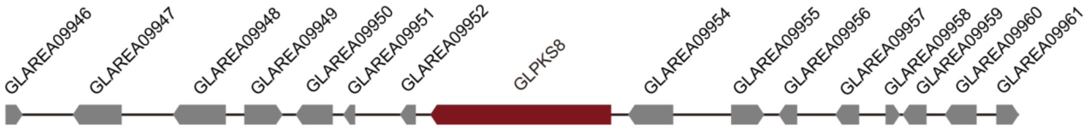

P-loop containing nucleoside triphosphate (GLAREA09946) MFS general substrate transporter (GLAREA09947, GLAREA09949)

Zn2/Cys6 DNA-binding (GLAREA09948) O-methyltransferase (GLAREA09950, GLAREA09961)

Alpha/beta-Hydrolases (GLAREA09951, GLAREA09952, GLAREA09956, GLAREA09959) Cytochrome P450 (GLAREA09954)

Hypothetical protein (GLAREA09955, GLAREA09957, GLAREA09960) Isochorismatase-like hydrolases (GLAREA09958)

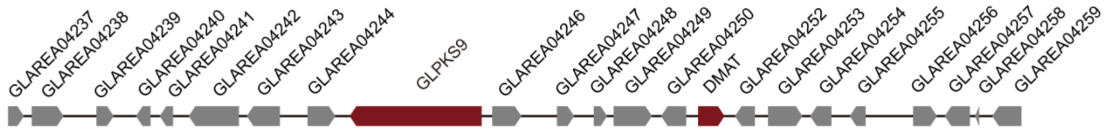

6-phosphogluconate dehydrogenase (GLAREA04237) Zn2/Cys6 DNA-binding (GLAREA04238) Isochorismatase-like (GLAREA04239)

Hypothetical protein (GLAREA04240, GLAREA04241, GLAREA04248, GLAREA04252, GLAREA04258, GLAREA04259)

MurD-like peptide ligases (GLAREA04242) MFS general substrate transporter (GLAREA04243, GLAREA04253)

Cytochrome P450 (GLAREA04244, GLAREA04246, GLAREA04254, GLAREA04257) O-methyltransferase (GLAREA04247, GLAREA04256)

FAD/NAD(P)-binding (GLAREA04249) Clavaminate synthase-like (GLAREA04250) NAD(P)-binding Rossmann-fold (GLAREA04255)

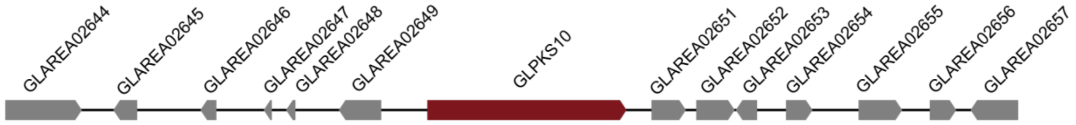

Protein kinase-like (GLAREA02644) MFS general substrate transporter (GLAREA02649) Glycosidases (GLAREA02651)

Hypothetical protein (GLAREA02645, GLAREA02646, GLAREA02647, GLAREA02648, GLAREA02653, GLAREA02654, GLAREA02657)

Arabinanase/levansucrase/invertase (GLAREA02652) Six-hairpin glycosidases (GLAREA02655) WD40 repeat-like (GLAREA02656)

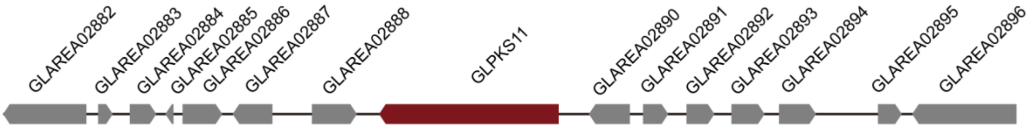

Cysteine proteinases (GLAREA02882) NADH-ubiquinone oxidoreductase (GLAREA02883)

hypothetical protein (GLAREA02884, GLAREA02885, GLAREA02887, GLAREA02888, GLAREA02891, GLAREA02895)

2,3-Bisphosphoglycerate-independent (GLAREA02886) MFS general substrate transporter (GLAREA02890)

Alpha/beta-Hydrolases (GLAREA02892) Cytochrome P450 (GLAREA02893, GLAREA02896) FAD/NAD(P)-binding (GLAREA02894)

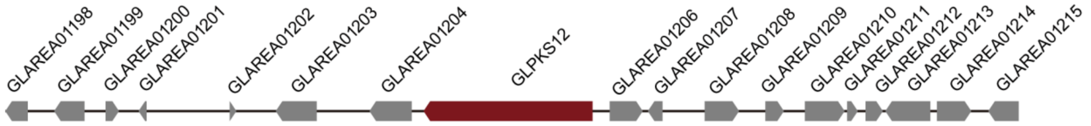

Hypothetical protein (GLAREA01198, GLAREA01199, GLAREA01200, GLAREA01201, GLAREA01203, GLAREA01204, GLAREA01207, GLAREA01215)

F-box (GLAREA01202) Clavaminate synthase-like (GLAREA01206) Cytochrome P450 (GLAREA01208, GLAREA01214)

NAD(P)-binding Rossmann-fold (GLAREA01209, GLAREA01212) FAD-binding (GLAREA01210) Dimeric alpha+beta barrel (GLAREA01211)

MFS general substrate transporter (GLAREA01213)

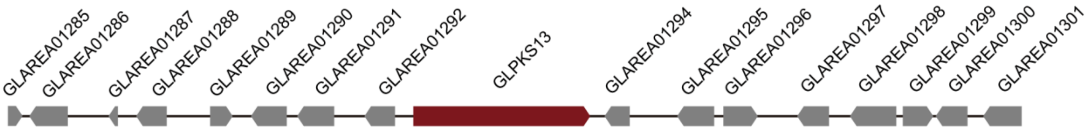

Hypothetical protein (GLAREA01285, GLAREA01286, GLAREA01288, GLAREA01289, GLAREA01290, GLAREA01292, GLAREA01295, GLAREA01300, GLAREA01301)

Glyoxalase/Bleomycin resistance (GLAREA01287) MFS general substrate transporter (GLAREA01291) Zn2/Cys6 DNA-binding (GLAREA01294)

Cytochrome P450 (GLAREA01296) UDP-Glycosyltransferase/glycogen phosphorylase (GLAREA01297) ARID-like (GLAREA01298)

DHHC zinc finger-containing protein (GLAREA01299)

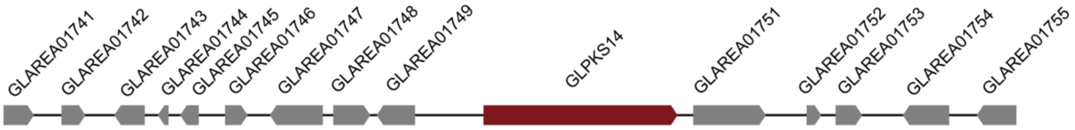

NAD(P)-binding Rossmann-fold (GLAREA01741, GLAREA01749) Histone-fold (GLAREA01742)

Hypothetical protein (GLAREA01743, GLAREA01744, GLAREA01752, GLAREA01753, GLAREA01755)

Pyrrolidone carboxyl peptidase (GLAREA01745) Protein kinase-like (GLAREA01746) Acetyl-CoA synthetase-like (GLAREA01747)

Thiolase-like (GLAREA01748) Zn2/Cys6 DNA-binding (GLAREA01751, GLAREA01754)

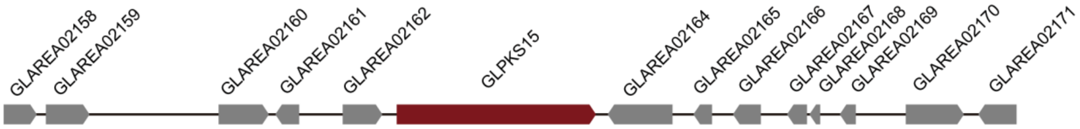

Hyprothetical protein (GLAREA02158, GLAREA02159, GLAREA02162, GLAREA02166, GLAREA02167, GLAREA02169, GLAREA02170, GLAREA02171)

MFS general substrate transporter (GLAREA02160) P-loop containing nucleoside triphosphate (GLAREA02161)

CorA soluble (GLAREA02164) NAD(P)-binding Rossmann-fold (GLAREA02165) FAD/NAD(P)-binding (GLAREA02168)

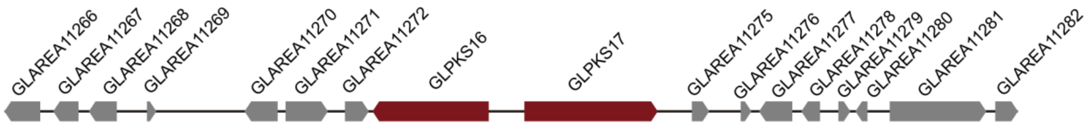

Hypothetical protein （GLAREA11266， GLAREA11267， GLAREA11268， GLAREA11269, GLAREA11275， GLAREA11277， GLAREA11279， GLAREA11282）

MFS general substrate transporter (GLAREA11270) Zn2/Cys6 DNA-binding (GLAREA11271) Protein kinase-like (GLAREA11272, GLAREA11278)

TPR-like (GLAREA11276) LysM (GLAREA11280) C2H2 and C2HC zinc fingers (GLAREA11281)

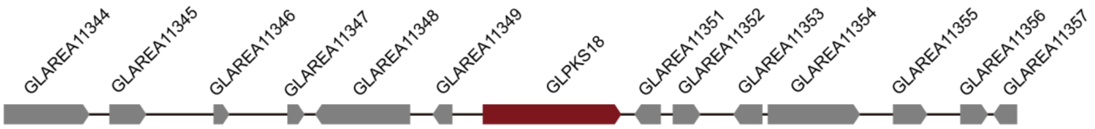

AF1104-like （GLAREA11344） Protein kinase-like (GLAREA11345， GLAREA11354， GLAREA11357)

Hypothetical protein (GLAREA11346， GLAREA11347， GLAREA11349， GLAREA11353) P-loop containing nucleoside triphosphate (GLAREA11348)

NAD kinase (GLAREA11351) Glycerol-3-phosphate (1)-acyltransferase (GLAREA11352) Cytochrome P450 (GLAREA11355)

ClpP/crotonase (GLAREA11356)

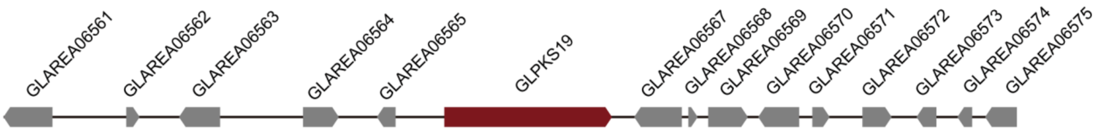

Hypothetical protein (GLAREA06561, GLAREA06562, GLAREA06567, GLAREA06570, GLAREA06572, GLAREA06573, GLAREA06574)

MFS general substrate transporter (GLAREA06563) Alkaline phosphatase-like (GLAREA06564)

Alpha/beta-Hydrolases (GLAREA06565) Sm-like ribonucleoproteins (GLAREA06568) AhpD-like (GLAREA06571)

C_2H_2 and C_2HC zinc fingers (GLAREA06575)

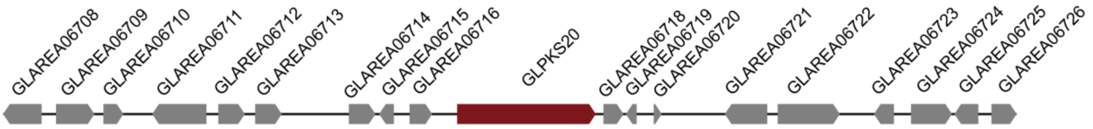

MFS general substrate transporter (GLAREA06708) FAD/NAD(P)-binding (GLAREA06709)

NAD(P)-binding Rossmann-fold (GLAREA06710, GLAREA06712，GLAREA06716) Thiamin diphosphate-binding fold (GLAREA06711)

Putative cyclase (GLAREA06713) Cytochrome P450 (GLAREA06714)NTF2-like (GLAREA06715) Protein kinase-like (GLAREA06718)

Hypothetical protein (GLAREA06719， GLAREA06720， GLAREA06721， GLAREA06724) Metallo-hydrolase/oxidoreductase (GLAREA06722)

Chaperone J-domain (GLAREA06723) Clavaminate synthase-like (GLAREA06725) Metallo-dependent hydrolases (GLAREA06726)

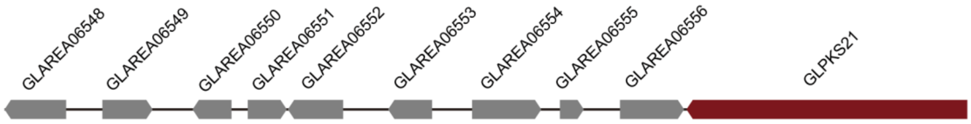

Hypothetical protein (GLAREA06548, GLAREA06549, GLAREA06554) Clavaminate synthase-like (GLAREA06550)
Galactose-binding (GLAREA06551) Metallo-dependent hydrolases (GLAREA06552) Nucleoside hydrolase (GLAREA06553)
Phosphoglycerate mutase-like (GLAREA06555) Acetyl-CoA synthetase-like (GLAREA06556)

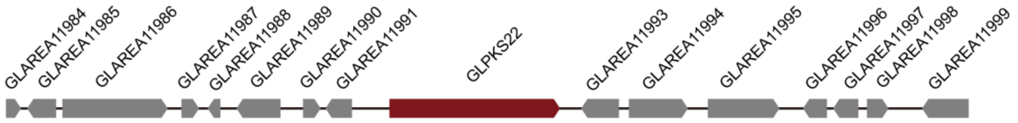

Alpha-L RNA-binding motif (GLAREA11984) Hypothetical protein (GLAREA11985, GLAREA11988, GLAREA11993, GLAREA11994, GLAREA11997)
DNA/RNA polymerases (GLAREA11986) NAD(P)-binding Rossmann-fold (GLAREA11987) MFS general substrate transporter (GLAREA11989)
Alpha/beta-Hydrolases (GLAREA11990) Zn2/Cys6 DNA-binding (GLAREA11991) I/LWEQ (GLAREA11995)
Alpha box protein (GLAREA11996) HMG-box (GLAREA11998) DNase I-like (GLAREA11999)

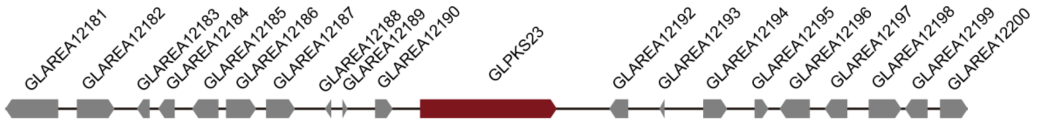

RING/U-box (GLAREA12181)
Predicted protein (GLAREA12182, GLAREA12184, GLAREA12185, GLAREA12186, GLAREA12187, GLAREA12189, GLAREA12192, GLAREA12193, GLAREA12200)
His-Me finger endonucleases (GLAREA12183) similar to tyrosinase central protein (GLAREA12188) Metallo-dependent hydrolases (GLAREA12190)
POZ (GLAREA12194, GLAREA12197) Concanavalin A-like lectins/glucanases (GLAREA12195) MFS general substrate transporter (GLAREA12196)
Winged helix DNA-binding (GLAREA12198) Alpha/beta-Hydrolases (GLAREA12199)

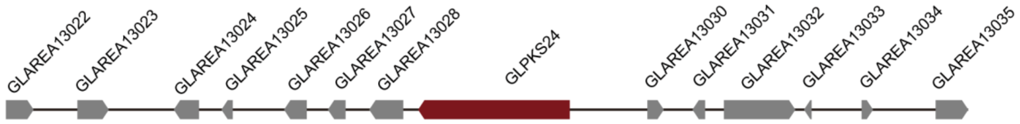

RING/U-box (GLAREA13022)
Hypothetical protein (GLAREA13023, GLAREA13025, GLAREA13027, GLAREA13028, GLAREA13030, GLAREA13031, GLAREA13032, GLAREA13033, GLAREA13034, GLAREA13035)
NAD(P)-binding Rossmann-fold (GLAREA13024) Soluble quinoprotein glucose dehydrogenase (GLAREA13026)

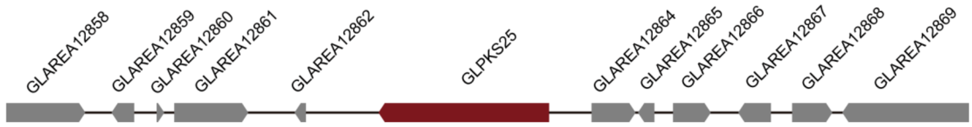

C_2H_2 and C_2HC zinc fingers (GLAREA12858) Protein kinase-like (GLAREA12859) Hypothetical protein (GLAREA12860, GLAREA12862, GLAREA12865)
WD40 repeat-like (GLAREA12861) Cytochrome P450 (GLAREA12864, GLAREA12867) MFS general substrate transporter (GLAREA12866, GLAREA12869)
GABA permease, putative (GLAREA12868)

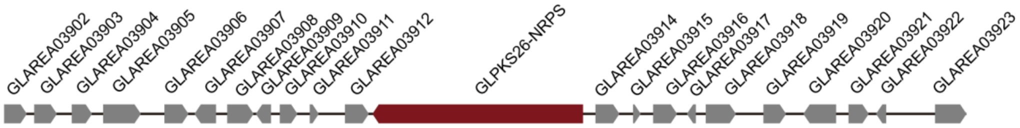

DmpA/ArgJ-like (GLAREA03902) Pc13g08980 (GLAREA03903)
Hypothetical protein (GLAREA03904, GLAREA03909, GLAREA03910, GLAREA03911, GLAREA03912, GLAREA03916, GLAREA03922)
P-loop containing nucleoside triphosphate (GLAREA03905) (Trans)glycosidases (GLAREA03906) WD40 repeat-like (GLAREA03907)
Clavaminate synthase-like (GLAREA03908) Alpha/beta-Hydrolases (GLAREA03914) Integral membrane protein (GLAREA03915, GLAREA03917)
Metallo-dependent hydrolases (GLAREA03918) Zn2/Cys6 DNA-binding (GLAREA03919) MFS general substrate transporter (GLAREA03920)
GroES-like (GLAREA03921) FAD-binding (GLAREA03923)

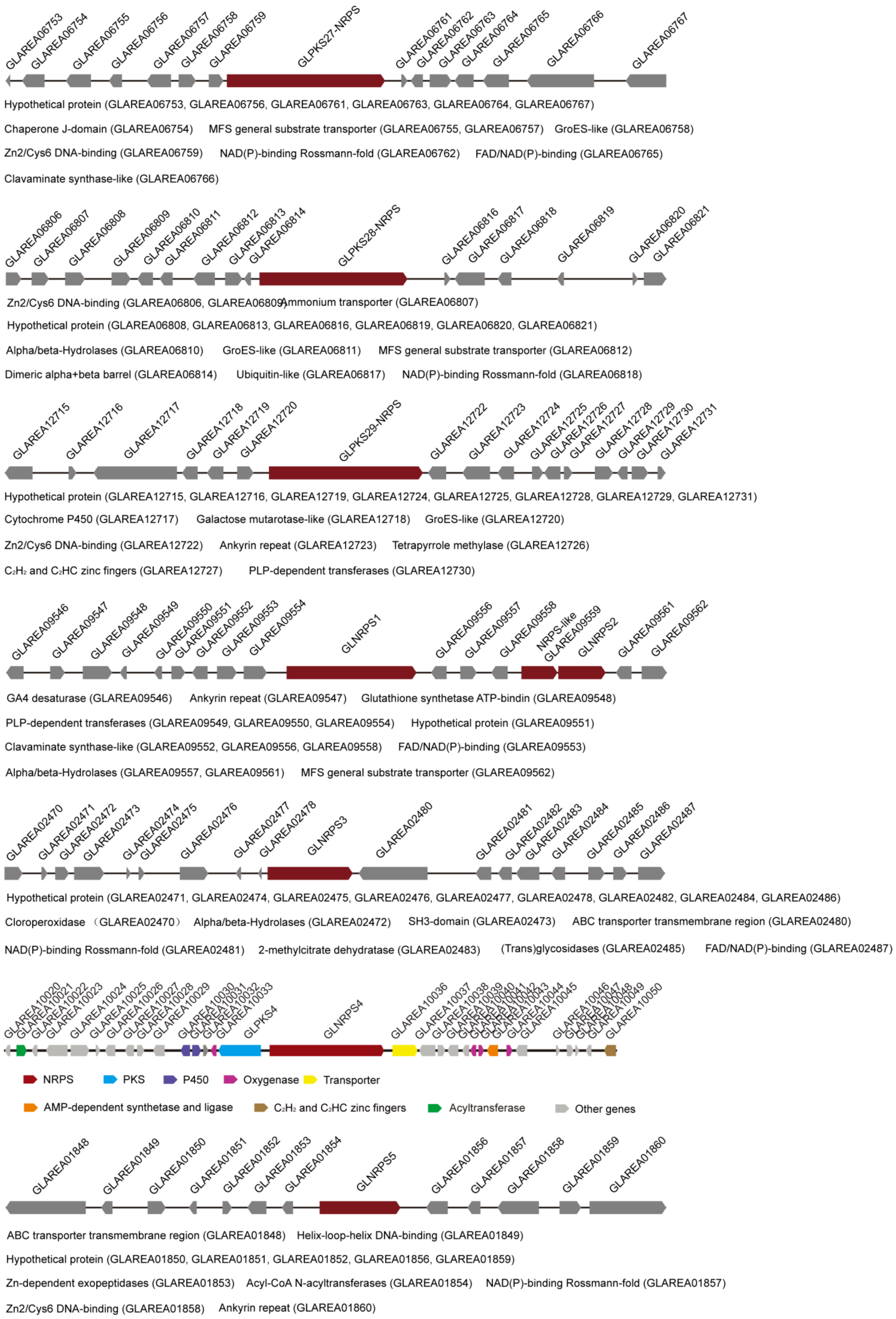
GLPKS27-NRPS
Hypothetical protein (GLAREA06753, GLAREA06756, GLAREA06761, GLAREA06763, GLAREA06764, GLAREA06767)
Chaperone J-domain (GLAREA06754)
MFS general substrate transporter (GLAREA06755, GLAREA06757)
GroES-like (GLAREA06758)
Zn2/Cys6 DNA-binding (GLAREA06759)
NAD(P)-binding Rossmann-fold (GLAREA06762)
FAD/NAD(P)-binding (GLAREA06765)
Clavaminate synthase-like (GLAREA06766)
GLPKS28-NRPS
Zn2/Cys6 DNA-binding (GLAREA06806, GLAREA06809)
Ammonium transporter (GLAREA06807)
Hypothetical protein (GLAREA06808, GLAREA06813, GLAREA06816, GLAREA06819, GLAREA06820, GLAREA06821)
Alpha/beta-Hydrolases (GLAREA06810)
GroES-like (GLAREA06811)
MFS general substrate transporter (GLAREA06812)
Dimeric alpha+beta barrel (GLAREA06814)
Ubiquitin-like (GLAREA06817)
NAD(P)-binding Rossmann-fold (GLAREA06818)
GLPKS29-NRPS
Hypothetical protein (GLAREA12715, GLAREA12716, GLAREA12719, GLAREA12724, GLAREA12725, GLAREA12728, GLAREA12729, GLAREA12731)
Cytochrome P450 (GLAREA12717)
Galactose mutarotase-like (GLAREA12718)
GroES-like (GLAREA12720)
Zn2/Cys6 DNA-binding (GLAREA12722)
Ankyrin repeat (GLAREA12723)
Tetrapyrrole methylase (GLAREA12726)
C₂H₂ and C₂HC zinc fingers (GLAREA12727)
PLP-dependent transferases (GLAREA12730)
GLNRPS1
NRPS-like
GLNRPS2
GA4 desaturase (GLAREA09546)
Ankyrin repeat (GLAREA09547)
Glutathione synthetase ATP-bindin (GLAREA09548)
PLP-dependent transferases (GLAREA09549, GLAREA09550, GLAREA09554)
Hypothetical protein (GLAREA09551)
Clavaminate synthase-like (GLAREA09552, GLAREA09556, GLAREA09558)
FAD/NAD(P)-binding (GLAREA09553)
Alpha/beta-Hydrolases (GLAREA09557, GLAREA09561)
MFS general substrate transporter (GLAREA09562)
GLNRPS3
Hypothetical protein (GLAREA02471, GLAREA02474, GLAREA02475, GLAREA02476, GLAREA02477, GLAREA02478, GLAREA02482, GLAREA02484, GLAREA02486)
Cloroperoxidase （GLAREA02470）
Alpha/beta-Hydrolases (GLAREA02472)
SH3-domain (GLAREA02473)
ABC transporter transmembrane region (GLAREA02480)
NAD(P)-binding Rossmann-fold (GLAREA02481)
2-methylcitrate dehydratase (GLAREA02483)
(Trans)glycosidases (GLAREA02485)
FAD/NAD(P)-binding (GLAREA02487)
GLPKS4
GLNRPS4
NRPS
PKS
P450
Oxygenase
Transporter
AMP-dependent synthetase and ligase
C₂H₂ and C₂HC zinc fingers
Acyltransferase
Other genes
GLNRPS5
ABC transporter transmembrane region (GLAREA01848)
Helix-loop-helix DNA-binding (GLAREA01849)
Hypothetical protein (GLAREA01850, GLAREA01851, GLAREA01852, GLAREA01856, GLAREA01859)
Zn-dependent exopeptidases (GLAREA01853)
Acyl-CoA N-acyltransferases (GLAREA01854)
NAD(P)-binding Rossmann-fold (GLAREA01857)
Zn2/Cys6 DNA-binding (GLAREA01858)
Ankyrin repeat (GLAREA01860)

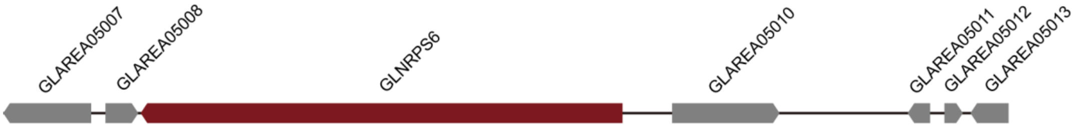

Acetylornithine deacetylase (GLAREA05007)   MFS general substrate transporter (GLAREA05008)

P-loop containing nucleoside triphosphate (GLAREA05010)   Hypothetical protein (GLAREA05011, GLAREA05012, GLAREA05013)

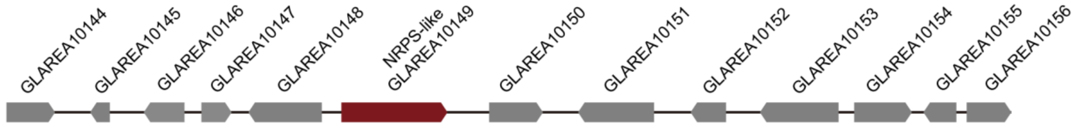

MFS general substrate transporter (GLAREA10144, GLAREA10150)   Class I glutamine amidotransferase-like (GLAREA10145)

Hypothetical protein (GLAREA10146, GLAREA10154)   Alpha/beta-Hydrolases (GLAREA10147)

P-loop containing nucleoside triphosphate (GLAREA10148, GLAREA10151)   MFS general substrate transporter (GLAREA10150)

Mitochondrial carrier (GLAREA10152)   Autophagy-related protein (GLAREA10153)   6-phosphogluconate dehydrogenase (GLAREA10155)

NAD(P)-binding Rossmann-fold (GLAREA10156)

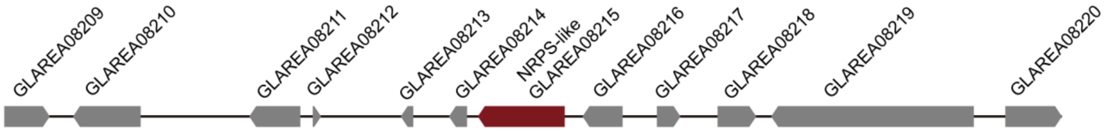

Hypothetical protein (GLAREA08209, GLAREA08210, GLAREA08212, GLAREA08213)

Family A G protein-coupled receptor-like (GLAREA08211)   DHH phosphoesterases (GLAREA08214)

Dehydroquinate synthase-like (GLAREA08216)   Methyltransferase (GLAREA08217)   Glutathione synthetase ATP-binding (GLAREA08218)

ARM repeat (GLAREA08219)   Riboflavin synthase (GLAREA08220)

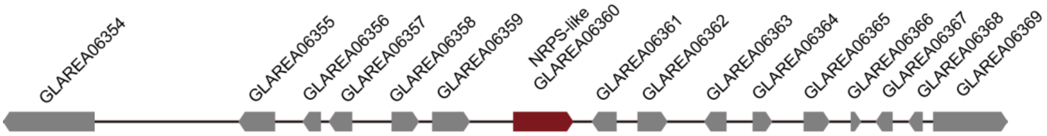

Ankyrin repeat (GLAREA06354, GLAREA06361, GLAREA06363, GLAREA06368)   Cytochrome P450 (GLAREA06355)

Hypothetical protein (GLAREA06356, GLAREA06357, GLAREA06359, GLAREA06362, GLAREA06366)

Concanavalin A-like lectins/glucanases (GLAREA06358)   C2H2 and C2HC zinc fingers (GLAREA06364)   Pectin lyase-like (GLAREA06365)

Protein kinase-like (GLAREA06367)   Actin-like ATPase (GLAREA06369)

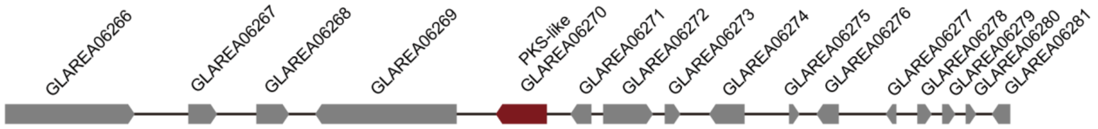

Hypothetical protein (GLAREA06266, GLAREA06267, GLAREA06273, GLAREA06274, GLAREA06275, GLAREA06276, GLAREA06277, GLAREA06278, GLAREA06279, GLAREA06280)

Concanavalin A-like lectins/glucanases (GLAREA06268)   NAD(P)-binding Rossmann-fold (GLAREA06269)   TLC-containing protein (GLAREA06271)

MFS general substrate transporter (GLAREA06272)   SET (GLAREA06281)

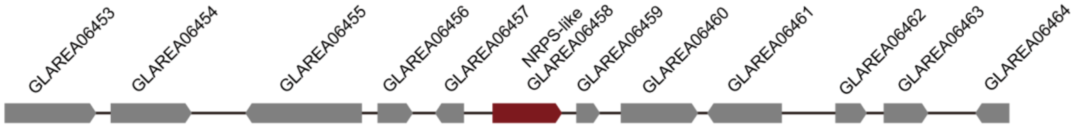

UBC-like (GLAREA06453)   ARM repeat (GLAREA06454)   Cytochrome P450 (GLAREA06455)   O-methyltransferase (GLAREA06456, GLAREA06464)

Hypothetical protein (GLAREA06457, GLAREA06463)   Alpha/beta-Hydrolases (GLAREA06459, GLAREA06462)

P-loop containing nucleoside triphosphate (GLAREA06460)   Class II aaRS and biotin synthetases (GLAREA06461)

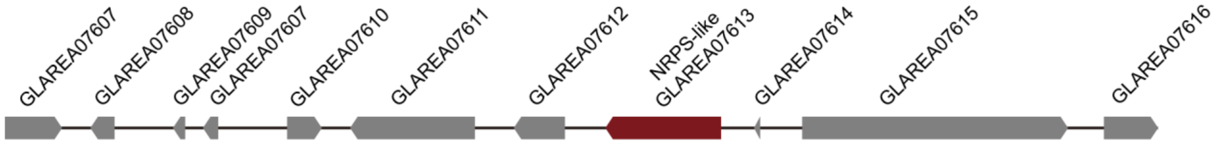

Class II aaRS and biotin synthetases (GLAREA07607)

Hypothetical protein (GLAREA07608, GLAREA07609, GLAREA07610, GLAREA07615) Aquaporin-like (GLAREA07611)

Zn2/Cys6 DNA-binding (GLAREA07612) MFS general substrate transporter (GLAREA07613)

P-loop containing nucleoside triphosphate (GLAREA07616) S-adenosyl-L-methionine-dependent (GLAREA07617)

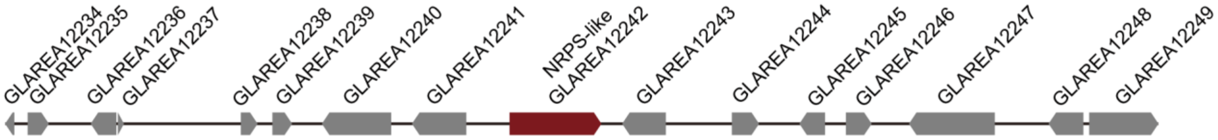

Hypothetical protein (GLAREA12234, GLAREA12236, GLAREA12237, GLAREA12244, GLAREA12245, GLAREA12247)

Methyltransferase (GLAREA12235) Ubiquitin-like (GLAREA12238) Cap-Gly (GLAREA12239) Alpha/beta-Hydrolases (GLAREA12240)

NCS1 nucleoside transporter (GLAREA12241) RING/U-box (GLAREA12243) P-loop containing nucleoside triphosphate (GLAREA12246)

WD40 repeat-like (GLAREA12248, GLAREA12249)

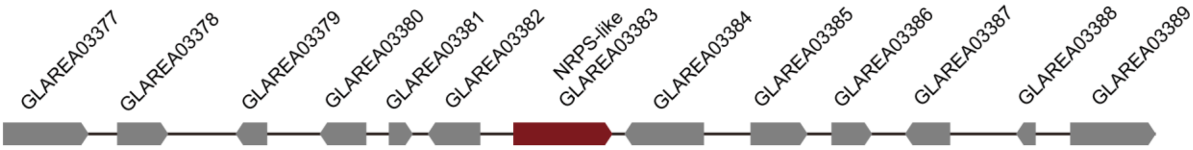

C2H2 and C2HC zinc fingers (GLAREA03377) MFS general substrate transporter (GLAREA03378)

Hypothetical protein (GLAREA03379, GLAREA03382, GLAREA03385, GLAREA03386, GLAREA03387, GLAREA03388)

6-phosphogluconate dehydrogenase (GLAREA03380) SET (GLAREA03381) Six-hairpin glycosidases (GLAREA03384)

F-box (GLAREA03389)

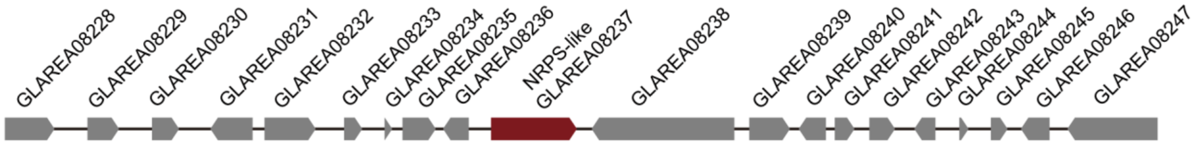

Hypothetical protein (GLAREA08228, GLAREA08229, GLAREA08230, GLAREA08231, GLAREA08232, GLAREA08233, GLAREA08234, GLAREA08235 GLAREA08236, GLAREA08240, GLAREA08242, GLAREA08243, GLAREA08244, GLAREA08245, GLAREA08246)

Sec7 (GLAREA08238) CoA-dependent acyltransferases (GLAREA08239) Thiamin pyrophosphokinase, catalytic (GLAREA08241)

Cysteine proteinases (GLAREA08247)

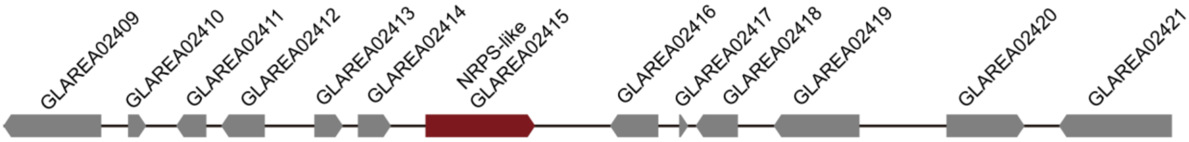

Alpha/beta-Hydrolases (GLAREA02409) Acyl-CoA N-acyltransferases (GLAREA02410)

Hypothetical protein (GLAREA02411, GLAREA02414, GLAREA02420) Zn-dependent exopeptidases (GLAREA02412)

Methyltransferase (GLAREA02413) Cytochrome P450 (GLAREA02416) NAD(P)-binding Rossmann-fold (GLAREA02417)

(Trans)glycosidases (GLAREA02418) Subtilisin-like (GLAREA02419) HCP-like (GLAREA02421)

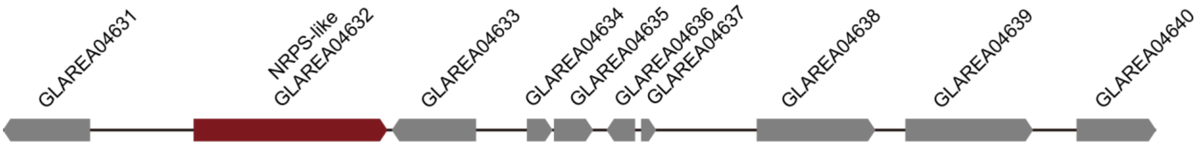

PLP-dependent transferases (GLAREA04631) FAD-binding (GLAREA04633) MFS general substrate transporter (GLAREA04634, GLAREA04635)

NAD(P)-binding Rossmann-fold (GLAREA04636) Hypothetical protein (GLAREA04637, GLAREA04638, GLAREA04640)

C-terminal (heme d1) of cytochrome cd1-nitrite (GLAREA04639)

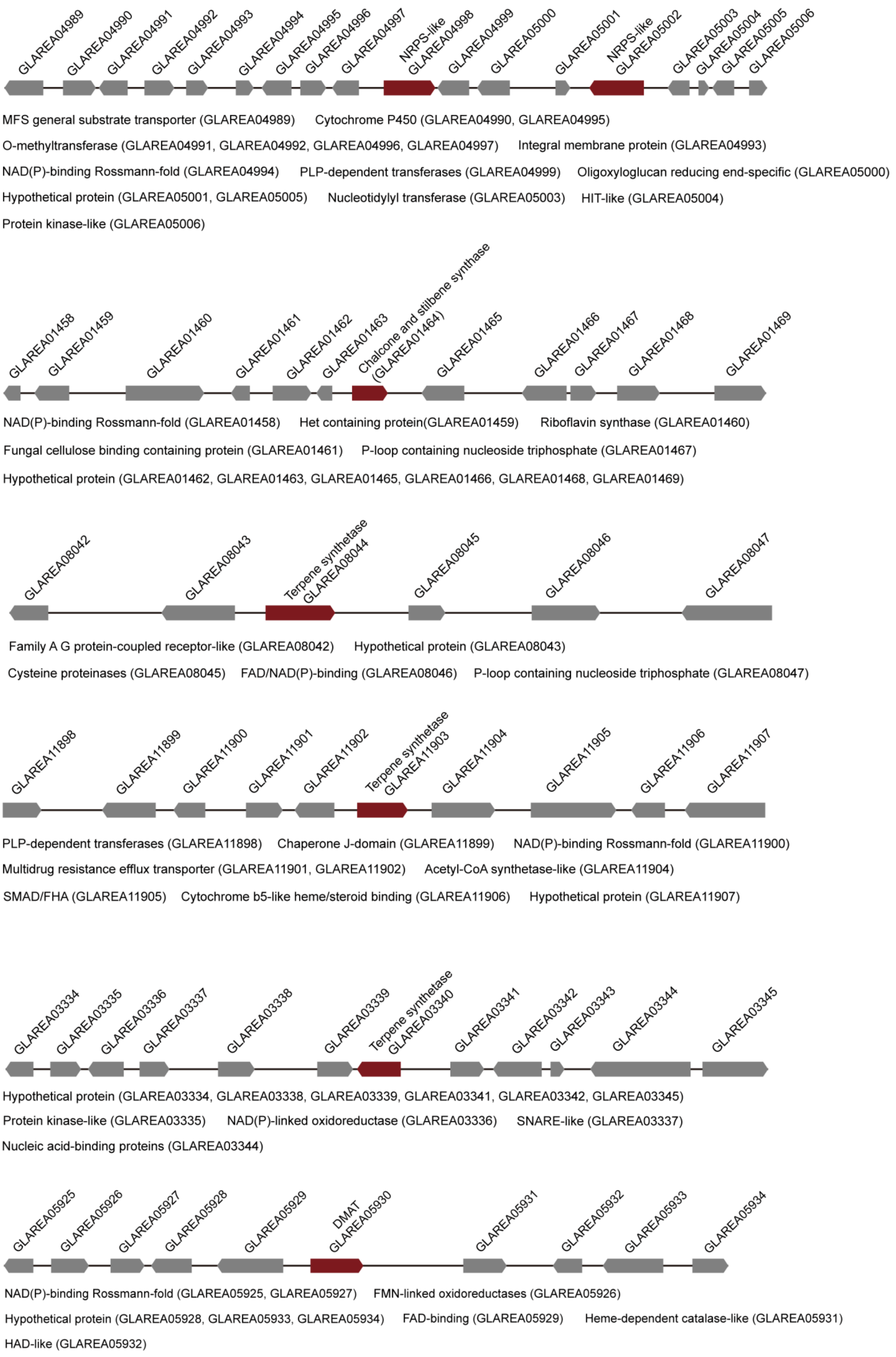

Figure S1. The 49 putative and proven secondary metabolite biosynthetic gene clusters in the *G. lozoyensis* genome.

(a)

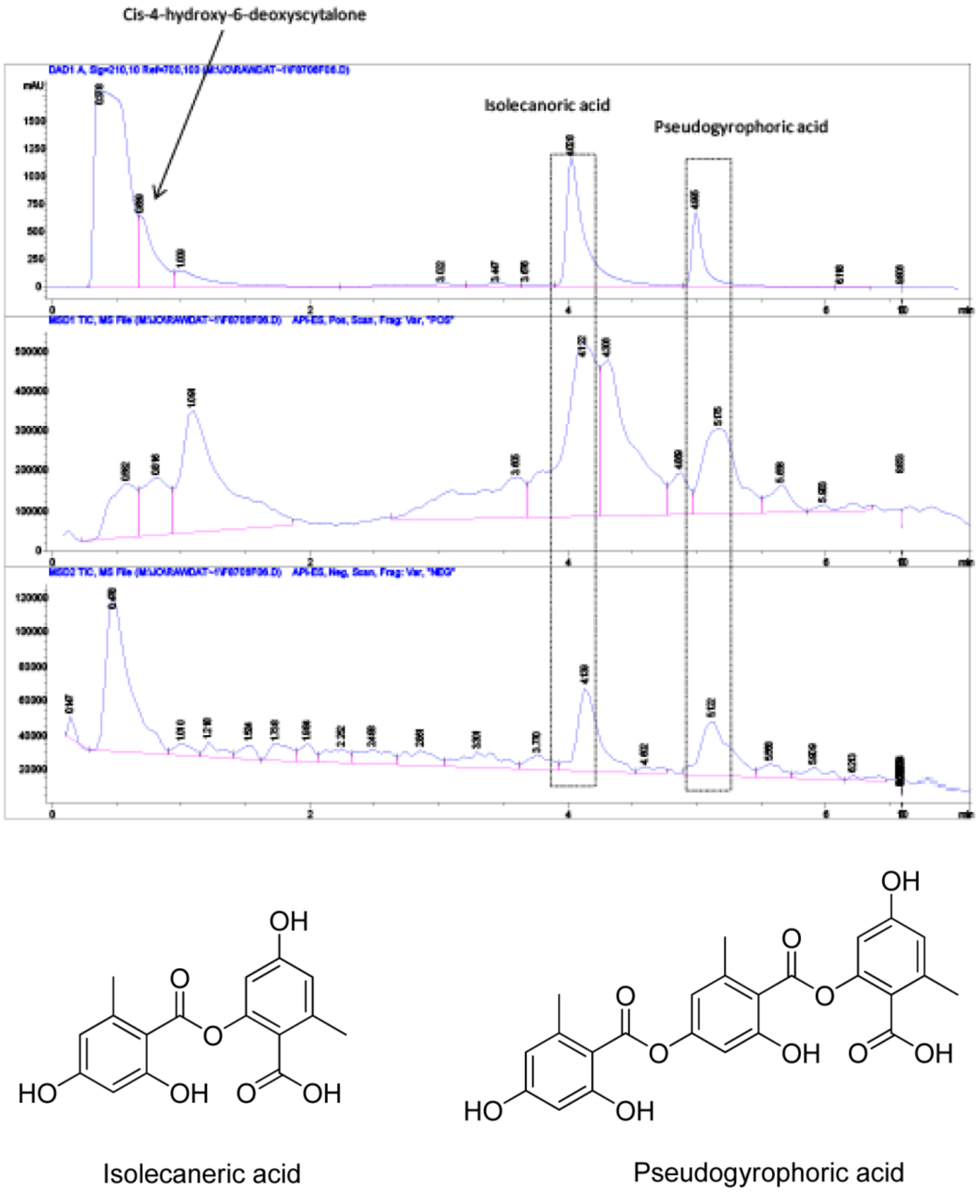

(b)

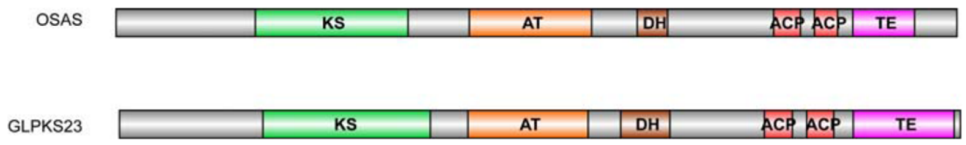

Figure S2. Materials and methods for purification and characterization of additional metabolites from *G. lozoyensis* ATCC 20868 grown on MV8 medium. Standard HPLC-MS data were obtained with an Agilent 1100 single quadrupole HPLC-MS chromatograph, with a stationary phase Phenomenex Luna (C18, 2 × 50 mm) maintained at 40 ºC. A mixture of two solvents, A (10% acetronitrile, 90% water) and B (90% acetronitrile, 10% water), both containing 1.3 mM trifluoroacetic acid and 1.3 mM ammonium formate, was used as the mobile phase under a linear-gradient elution mode (B, 10-100%, 10 min) at a flow rate of 0.3 mL min^{-1}. Ionization of the eluting solvent was obtained using the standard Agilent 1100 ESI source adjusted to

a drying gas flow of 11 L min^{-1} at 325 ºC and a nebulizer pressure of 40 psi. The capillary voltage was set to 3,500 V. Mass spectra were collected as full scans from 150 *m/z* to 1,500 *m/z*, with one scan every 0.77 second, in both positive and negative modes. HR-MS was performed with an ESI-TOF Maxis Bruker HPLC-MS, operating in positive ESI mode (capillary voltage: 4 kV). (a) HPLC and mass spectra data for acetone extracts of *G. lozoyensis* indicating the major metabolites, isolecanoric acid and pseudogyrophoric acid. (b) Protein domain architecture of GLPKS23 from *G. lozoyensis* and OSAS from *A. nidulans*. KS, ketosynthase domain; AT, acyltransferase domain; DH, dehydratase domain; ACP, acyl carrier protein domain; TE, thioesterase domain.

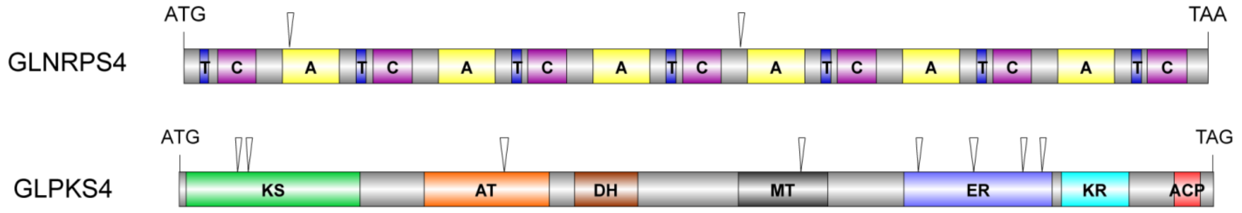

Figure S3. Gene structures of GLNRPS4 and GLPKS4. Triangles represent introns. A, adenylation domain; T, thiolation domain; C, condensation domain; KS, ketosynthase domain; AT, acyltransferase domain; DH, dehydratase domain; MT, methyltransferase domain; ER, enoylreductase domain; KR, β-ketoacylreductase domain; ACP, acyl carrier protein domain.

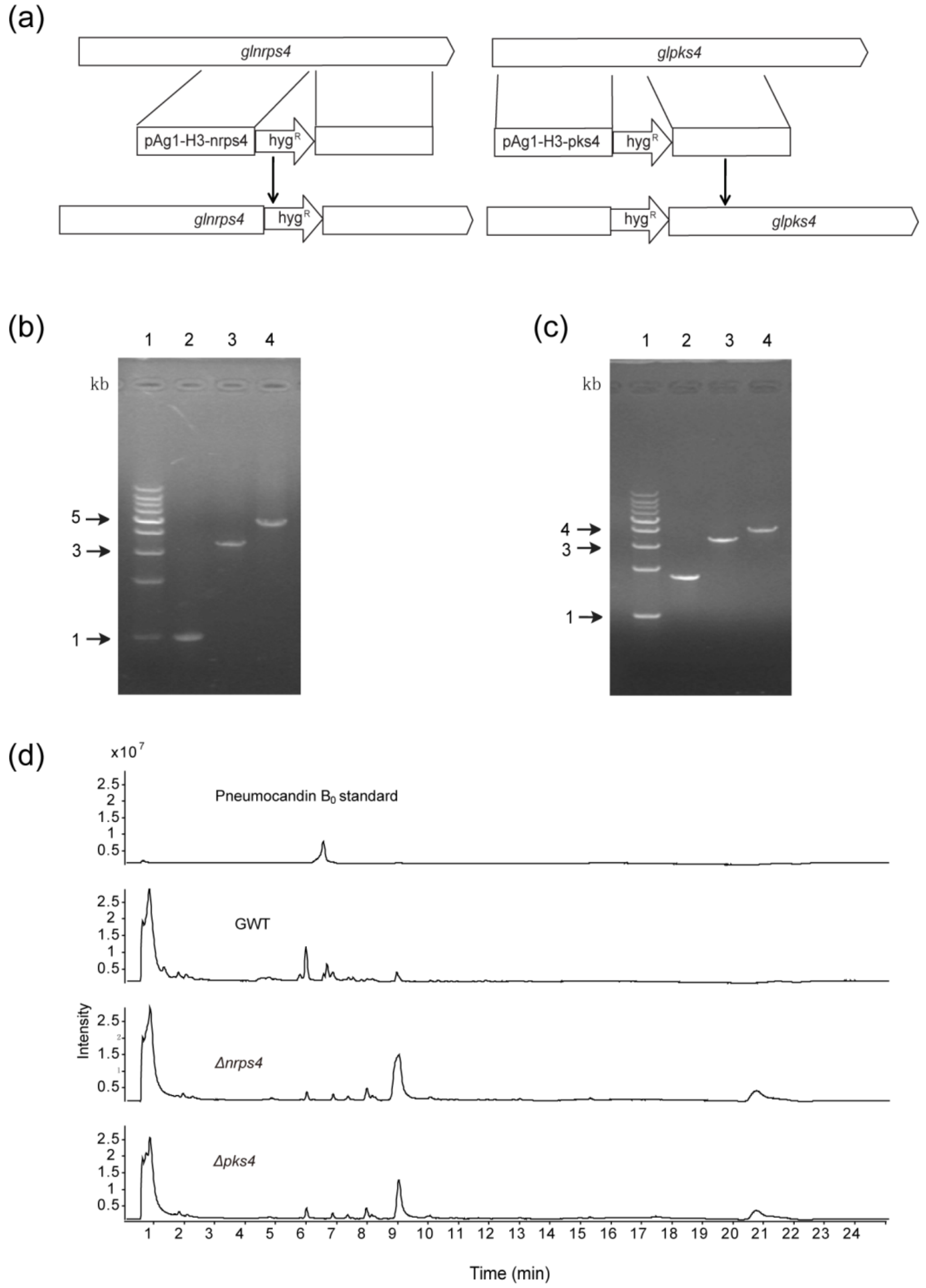

Figure S4. Strategy for the construction of GLPKS4 and **GLNRPS4 gene-deletion mutants.** (a) Schematic diagram of gene-deletion constructs for GLPKS4 and GLNRPS4 . (b) PCR detection for positive mutants of *Δglnrps4*. 1, DNA marker; 2–3 show PCR products amplified by primers C and D (**Table S5**). 2, wild-type (WT) strain; 3, positive mutant; 4, PCR products from positive mutant genome amplified by primers E and F (**Table S5**). (c) PCR detection for positive mutants of *Δglpks4*. 1, DNA marker; 2–3 show PCR products amplified by primers G and H (**Table S5**). 2, WT strain; 3, positive mutant; 4, PCR products from positive mutant genome amplified by primers I and J. (d) Original HPLC-MS profiles (TIC, + mode) of *Δglnrps4* and *Δglpks4,* WT, and pneumocandin B_0 standard.
