# Supplementary material for: Genomics-driven discovery of the pneumocandin biosynthetic gene cluster in the fungus Glarea lozoyensis
Source: BMC Genomics. 2013 May 20;14:339. doi: 10.1186/1471-2164-14-339 (PMC3672099; doi:10.1186/1471-2164-14-339)
Supplement: Additional file 2 — Tables that provide support information for the main text. Table S1 shows the homologous analysis of terpene gene in Glarea lozoyensis. Table S2 lists the fungal genomes used for phylogenomic analyses and CAZymes analysis. Table S3 provides supporting data for Figure 6 and the list of 71 functionally characterized fungal PKSs and PKS-NRPSs hydrids, their gene designations, the principal pathway end product, and references. Table S4 shows polyketide synthases (PKSs) in the G. lozoyensis genome used for phylogenetic tree construction. Table S5 summarizes primers used for genes deletion and mutants verification. [file 1471-2164-14-339-S2.pdf]

Table S1. Terpene synthease genes in *G. lozoyensis*

Gene ID	Putative function	Closest similarity (identifity/accession number/Fungus)
GLAREA03340	geranylgeranyl pyrophosphate synthase	78%/CCD33657.1/*Botryotinia fuckeliana*
GLAREA04679	farnesyl pyrophosphate synthetase	89%/EKD14727.1/*Marssonina brunnea*
GLAREA04931	geranylgeranyl transferase	78%/CCD56919.1/*Botryotinia fuckeliana*
GLAREA02940	farnesyl transferase	65%/CCD51427.1/*Botryotinia fuckeliana*
GLAREA01968	Squalene/phytoene synthase	56%/EKG16526.1/*Macrophomina phaseolina*
GLAREA02045	prenyltransferase and squalene oxidase	61%/EKD15616.1/*Marssonina brunnea*
GLAREA08044	Phytoene synthase/ lycopene cyclase	67%/EKD21546.1/*Marssonina brunnea*
GLAREA10578	geranylgeranyl pyrophosphate synthase	32%/GAA84687.1/*Aspergillus kawachii*
GLAREA06531	lanosterol synthase	81%/EKD19457.1/*Marssonina brunnea*
GLAREA07665	decaprenyl-diphosphate synthase	84%/CCD55693.1/*Botryotinia fuckeliana*
GLAREA11903	squalene synthetase	70%/EKD12656.1/*Marssonina brunnea*
GLAREA12472	ent-kaurene synthase	36%/XP_002849529.1/*Arthroderma otae*
GLAREA00660*	-	-
GLAREA11283*	-	-

*Identifity lower than 30% compared with the sequences deposited in NCBI database.

Table S2. The fungal genomes used for phylogenomic analyses and CAZymes analyses

Species	Data sources
Ascocoryne sarcoide	Prof. Scott A. Strobel,Yale University, New Haven, Connecticut, USA
Botrytis cinerea	http://www.broadinstitute.org/annotation/genome/botrytis_cinerea/
Sclerotinia sclerotiorm	http://www.broadinstitute.org/annotation/genome/sclerotinia_sclerotiorum/MultiHome.html
Blumeria graminis	NCBI accession: ABSB00000000
Magnaporthe oryzae	NCBI accession: AACU00000000
Aspergillus nidulans	NCBI accession: AACD00000000
Neurospora crassa	NCBI accession: AABX00000000
Fusarium graminearum	NCBI accession: AACM00000000
Epichloë festucae	http://www.endophyte.uky.edu/ef/
Verticillium albo-atrum	http://www.broadinstitute.org/annotation/genome/verticillium_dahliae/MultiHome.html
Saccharomyces cerevisiae	http://www.broadinstitute.org/annotation/genome/saccharomyces_cerevisiae
Trichoderma reesei	NCBI accession: AAIL00000000
Glomerella graminicola	NCBI accession: ACOD00000000
Tuber melanosporum	NCBI accession: CABJ00000000
Laccaria bicolor	NCBI accession: ABFE00000000
Piriformospora indica	NCBI accession: CAFZ00000000

Table S3. Reference polyketide synthases (PKSs) and fatty acid synthase (FAS) used for ketosynthase (KS) domain phylogenetic tree construction (Figure 6), their gene designations, end product metabolites, and references.

Species	PKS Name/Product	References
Alternaria alternata	ALM1/Melanin	[1]
Alternaria solani	Sol1/Solanapyrone	[2]
Alternaria solani	PKSN/Alternapyrone	[3]
Ascochyta rabiei	ArPKS1/1,8-dihydroxynaphthalene	[4]
Aspergillus flavus	CpaS/Cyclopiazonic acid	[5]
Aspergillus fumigatus	PsoA/Pseurotin A	[6]
Aspergillus fumigatus	Alb1/AFALB1/Dihydroxynaphthalene	[7]
Aspergillus fumigatus	encA/Endocrocin	[8]
Aspergillus nidulans	OrsA/Orsellinic acid	[9]
Aspergillus nidulans	mdpG/Anthraquinone	[9]
Aspergillus nidulans	WA/Naphthopyrone	[10]
Aspergillus nidulans	/STCA/ANST/Sterigmatocystin	[11]
Aspergillus nidulans	ApdA/Aspyridone	[12]
Aspergillus nidulans	ausA/Austinol	[13]
Aspergillus nidulans	EasB/ Emericellamide	[14]
Aspergillus niger	azaA/Azanigerones A–F	[15]
Aspergillus niger	azaB/Azanigerones A–F	[15]
Aspergillus niger	/AlbA/albA/Naphtho-γ-pyrones	[16]
Aspergillus parasiticus	/NSAS/pksL1/Norsolorinic acid	[17]
Aspergillus parasiticus	PKSL2/pksL2/6-methylsalicylic acid	[18]
Aspergillus oryzae	AoiG/aoiG/Methylated derivative of orthosporin	[19]
Aspergillus terreus	ATX/ATATX/6-methylsalicylic acid	[20]
Aspergillus terreus	LNKS/LovF/Lovastatin	[21]
Aspergillus terreus	LDKS/LovB/Lovastatin	[21]
Aspergillus terreus	ACAS/Emodin	[22]
Aspergillus terreus	Trt4/trt4/Terretonin	[23]
Aspergillus westerdijkiae	AoKS1/aoks1/Ochratoxin	[24]
Aspergillus westerdijkiae	aomsas/Isoasperlactone, asperlactone	[25]
Beauveria bassiana	TenS/2-Pyridone tenellin	[26]

Botryotinia fuckeliana	BcBOA9/Botcinic acid	[27,28]
Botryotinia fuckeliana	BcBOA9/ Botcinic acid	[27]
Ceratocystis resinifera	PKS1/1,3,6,8,-tetrahydroxynaphthalene	[29]
Cercospora nicotianae	CTB1/Cercosporin	[30]
Chaetomium chiversii	CcRADS/Radicicol	[31]
Chaetomium globosum	PKS1/Melanin	[32]
Chaetomium globosum	cazF/Chaetomugilin, chaetoviridin	[33]
Chaetomium globosum	cazM/Chaetomugilin, chaetoviridin	[33]
Cladonia metacorallifera	CmPKS1/6-methylsalicylic acid?	[34]
Cochliobolus heterostrophus	PKS1/T-toxin	[35]
Colletotrichum lagenarium	CLPKS1/1,3,6,8-tetrahydroxynaphthalene	[36]
Dothistroma septosporum	PksA/pksA/Norsolorinic acid	[37]
Elsinoë fawcettii	efpks1/Elsinochrome	[38]
Fusarium heterosporum	EqiS/Equisetin	[39]
Gibberella fujikuroi	PKS4/GFPKS4/Bikaverin	[40]
Gibberella fujikuroi	FUM5/GFFUM5/Fumonisin	[41]
Gibberella fujikuroi	fsr1/Fusarubins	[42]
Gibberella fujikuroi	fusA/Fusarin C	[43]
Gibberella moniliformis	Fub1/Fusaric acid	[44]
Gibberella zeae	PKS12/Aurofusarin	[45]
Gibberella zeae	FSL1/Fusarielins	[46]
Gibberella zeae	Pks13/Zearalenone	[47]
Glarea lozoyensis	GLPKS1/Tetrahydroxynaphthalene	[48]
Glarea lozoyensis	GLPKS4/Dimethyl-myristic acid	[49]
Glarea lozoyensis	GLPKS2/6-methylsalicylic acid	[50]
Hypomyces subiculosus.	Hmp3/Hypothemycin	[51]
Hypomyces subiculosus.	Hmp8/Hypothemycin	[51]
Hypoxylon pulicicidum	NSPKS1/Dihydroxynaphthalene	[52]
Metarhizium robertsii	NGS1/NG-391	[53]
Monascus purpureus	PksCT/Citrinin	[54]
Penicillium aethiopicum	GsfA/Griseofulvin	[55]
Penicillium aethiopicum	vrtA/Viridicatumtoxin	[55]

Penicillium brevicompactum	MpaC/5-methylorsellinic acid	[56]
Penicillium expansum	CheA/Chaetoglobosin A	[57]
Pencillium griseofulvum	WA type ketosynthase	[58]
Pencillium griseofulvum	PKS2/6-methylsalicylic acid	[59]
Penicillium nordicum	otapksPN/Ochratoxin	[60]
Penicillium patulum	PPMSAS/6-methylsalicylic acid	[61]
*Phoma sp.*C2932	phPKS1/Squalestatin side-chain tetraketide	[62]
Pochonia chlamydosporia	RDC1/Radicicol	[63]
Sarocladium strictum	MOS/3-methylorcinaldehyde	[64]
Sordaria macrospora	SMAC_03130/Dihydroxynaphthalene	[65]
Talaromyces marneffei	Pks11/Mitorubrinic acid, mitorubrinol	[66]
Talaromyces marneffei	Pks12/ Mitorubrinic acid, mitorubrinol	[66]
Talaromyces marneffei	alb1/Melanin	[67]
Talaromyces stipitatus	tropA/Stipitatic acid	[68]
Wangiella dermatitidis	WdPKS1/1,3,6,8-tetrahydroxynaphthalene	[69]
Xylaria sp. BCC 1067	PKS3/Xyrrolin	[70]
Rattus norvegicus	FAS/fatty acid	NP 059028

References for Figure 6 and Table S3

1. Kheder AA, Akagi Y, Akamatsu H, Yanaga K, Maekawa N, Otani H, Tsuge T, Kodama M: **Functional analysis of the melanin biosynthesis genes ALM1 and BRM2-1 in the tomato pathotype of *Alternaria alternata***. *J Gen Plant Pathol* 2012, **78**:30-38.
2. Kasahara K, Miyamoto T, Fujimoto T, Oguri H, Tokiwano T, Oikawa H, Ebizuka Y, Fujii I: **Solanapyrone synthase, a possible diels-alderase and iterative type I polyketide synthase encoded in a biosynthetic gene cluster from *Alternaria solani***. *ChemBioChem* 2010, **11**:1245-1252.
3. Fujii I, Yoshida N, Shimomaki S, Oikawa H, Ebizuka Y: **An iterative type I polyketide synthase PKSN catalyzes synthesis of the decaketide alternapyrone with regio-specific octa-methylation**. *Chem Biol* 2005, **12**:1301-1309.
4. Akamatsu HO, Chilvers MI, Stewart JE, Peever TL: **Identification and function of a polyketide synthase gene responsible for 1,8-dihydroxynaphthalene-melanin pigment biosynthesis in Ascochyta rabiei**. *Curr Genet* 2010, **56**:349-360.
5. Chang PK, Horn BW, Dorner JW: **Clustered genes involved in cyclopiazonic acid production are next to the aflatoxin biosynthesis gene cluster in *Aspergillus flavus***. *Fungal Genet Biol* 2009, **46**:176-182.
6. Maiya S, Grundmann A, Li X, Li SM, Turner G: **Identification of a hybrid PKS/NRPS required for pseurotin A biosynthesis in the human pathogen *Aspergillus fumigatus***. *ChemBioChem* 2007, **8**:1736-1743.
7. Tsai HF, Fujii I, Watanabe A, Wheeler MH, Chang YC, Yasuoka Y, Ebizuka Y, Kwon-Chung KJ: **Pentaketide melanin biosynthesis in *Aspergillus fumigatus* requires chain-length shortening of a heptaketide precursor**. *J Biol Chem* 2001, **276**:29292-29298.
8. Lim FY, Hou Y, Chen Y, Oh JH, Lee I, Bugni TS, Keller NP: **Genome-based cluster deletion reveals an endocrocin biosynthetic pathway in *Aspergillus fumigatus***. *Appl Environ Microbiol* 2012, **78**:4117-4125.

9. Scherlach K, Sarkar A, Schroeckh V, Dahse H-M, Roth M, Brakhage AA, Horn U, Hertweck C: **Two induced fungal polyketide pathways converge into antiproliferative spiroanthrones**. *ChemBioChem* 2011, **12**:1836-1839.
10. Watanabe A, Ebizuka Y: **A novel hexaketide naphthalene synthesized by a chimeric polyketide synthase composed of fungal pentaketide and heptaketide synthases**. *Tetrahedron Lett* 2002, **43**:843-846.
11. Yu JH, Leonard TJ: **Sterigmatocystin biosynthesis in *Aspergillus nidulans* requires a novel type I polyketide synthase**. *J Bacteriol* 1995, **177**:4792-4800.
12. Xu W, Cai X, Jung ME, Tang Y: **Analysis of intact and dissected fungal polyketide synthase-nonribosomal peptide synthetase in vitro and in *Saccharomyces cerevisiae***. *J Am Chem Soc* 2010, **132**:13604-13607.
13. Lo H-C, Entwistle R, Guo C-J, Ahuja M, Szewczyk E, Hung J-H, Chiang Y-M, Oakley BR, Wang CCC: **Two separate gene clusters encode the biosynthetic pathway for the meroterpenoids austinol and dehydroaustinol in *Aspergillus nidulans***. *J Am Chem Soc* 2012, **134**:4709-4720.
14. Chiang YM, Szewczyk E, Nayak T, Davidson AD, Sanchez JF, Lo HC, Ho WY, Simityan H, Kuo E, Praseuth A: **Molecular Genetic Mining of the *Aspergillus* Secondary Metabolome: Discovery of the Emericellamide Biosynthetic Pathway**. *Chem Biol* 2008, **15**:527-532.
15. Zabala AO, Xu W, Chooi YH, Tang Y: **Characterization of a silent azaphilone gene cluster from *Aspergillus niger* ATCC 1015 reveals a hydroxylation-mediated pyran-ring formation**. *Chem Biol* 2012, **19**:1049-1059.
16. Chiang Y-M, Meyer KM, Praseuth M, Baker SE, Bruno KS, Wang CCC: **Characterization of a polyketide synthase in *Aspergillus niger* whose product is a precursor for both dihydroxynaphthalene (DHN) melanin and naphtho-γ-pyrone**. *Fungal Genet Biol* 2011, **48**:430-437.
17. Ma Y, Smith LH, Cox RJ, Beltran-Alvarez P, Arthur CJ, Simpson TJ: **Catalytic relationships between type I and type II iterative polyketide synthases: The *Aspergillus parasiticus* norsolorinic acid synthase**. *ChemBioChem* 2006, **7**:1951-1958.
18. Feng GH, Leonard TJ: **Culture conditions control expression of the genes for aflatoxin and sterigmatocystin biosynthesis in *Aspergillus parasiticus* and *A. nidulans***. *Appl Environ Microbiol* 1998, **64**:2275-2277.
19. Nakazawa T, Ishiuchi K, Praseuth A, Noguchi H, Hotta K, Watanabe K: **Overexpressing transcriptional regulator in *Aspergillus oryzae* activates a silent biosynthetic pathway to produce a novel polyketide**. *ChemBioChem* 2012, **13**:855-861.
20. Fujii I, Ono Y, Tada H, Gomi K, Ebizuka Y, Sankawa U: **Cloning of the polyketide synthase gene atX from *Aspergillus terreus* and its identification as the 6-methylsalicylic acid synthase gene by heterologous expression**. *Mol Gen Genet* 1996, **253**:1-10.
21. Hendrickson L, Davis CR, Roach C, Nguyen DK, Aldrich T, McAda PC, Reeves CD: **Lovastatin biosynthesis in *Aspergillus terreus*: Characterization of blocked mutants, enzyme activities and a multifunctional polyketide synthase gene**. *Chem Biol* 1999, **6**:429-439.
22. Awakawa T, Yokota K, Funa N, Doi F, Mori N, Watanabe H, Horinouchi S: **Physically discrete β-lactamase-type thioesterase catalyzes product release in atrochrysone synthesis by iterative type I polyketide synthase**. *Chem Biol* 2009, **16**:613-623.
23. Guo C-J, Knox BP, Chiang Y-M, Lo H-C, Sanchez JF, Lee K-H, Oakley BR, Bruno KS, Wang CCC: **Molecular genetic characterization of a cluster in *A. terreus* for biosynthesis of the meroterpenoid terretonin**. *Org Lett* 2012, **14**:5684-5687.
24. Bacha N, Atoui A, Mathieu F, Liboz T, Lebrihi A: ***Aspergillus westerdijkiae* polyketide synthase gene "aoks1" is involved in the biosynthesis of ochratoxin A**. *Fungal Genet Biol* 2009, **46**:77-84.
25. Bacha N, Dao HP, Atoui A, Mathieu F, O'Callaghan J, Puel O, Liboz T, Dobson ADW, Lebrihi A: **Cloning and characterization of novel methylsalicylic acid synthase gene involved in the biosynthesis of isoasperlactone and asperlactone in *Aspergillus westerdijkiae***. *Fungal Genet Biol* 2009, **46**:742-749.
26. Halo LM, Heneghan MN, Yakasai AA, Song Z, Williams K, Bailey AM, Cox RJ, Lazarus CM, Simpson TJ: **Late stage oxidations during the biosynthesis of the 2-pyridone tenellin in the entomopathogenic fungus**

Beauveria bassiana. *J Am Chem Soc* 2008, **130**:17988-17996.
27. Dalmais B, Schumacher J, Moraga J, Le Pêcheur P, Tudzynski B, Collado IG, Viaud M: **The *Botrytis cinerea* phytotoxin botcinic acid requires two polyketide synthases for production and has a redundant role in virulence with botrydial**. *Mol Plant Pathol* 2011, **12**:564-579.
28. Massaroli M, Moraga J, Bastos Borges K, Ramírez-Fernández J, Viaud M, González Collado I, Durán-Patrón R, Hernández-Galán R: **A shared biosynthetic pathway for botcinins and botrylactones revealed through gene deletions**. *ChemBioChem* 2013, **14**:132-136.
29. Loppnau P, Tanguay P, Breuil C: **Isolation and disruption of the melanin pathway polyketide synthase gene of the softwood deep stain fungus *Ceratocystis resinifera***. *Fungal Genet Biol* 2004, **41**:33-41.
30. Choquer M, Dekkers KL, Chen HQ, Cao L, Ueng PP, Daub ME, Chung KR: **The CTB1 gene encoding a fungal polyketide synthase is required for cercosporin biosynthesis and fungal virulence of *Cercospora nicotianae***. *Mol Plant-Microbe Interact* 2005, **18**:468-476.
31. Wang S, Xu Y, Maine EA, Wijeratne EMK, Espinosa-Artiles P, Gunatilaka AAL, Molnár I: **Functional characterization of the biosynthesis of radicicol, an Hsp90 inhibitor resorcylic acid lactone from *Chaetomium chiversii***. *Chem Biol* 2008, **15**:1328-1338.
32. Hu Y, Hao XR, Lou J, Zhang P, Pan J, Zhu XD: **A PKS gene, pks-1, is involved in chaetoglobosin biosynthesis, pigmentation and sporulation in *Chaetomium globosum***. *Sci China Life Sci* 2012, **55**:1100-1108.
33. Winter JM, Sato M, Sugimoto S, Chiou G, Garg NK, Tang Y, Watanabe K: **Identification and characterization of the chaetoviridin and chaetomugilin gene cluster in *Chaetomium globosum* reveal dual functions of an iterative highly-reducing polyketide synthase**. *J Am Chem Soc* 2012, **134**:17900-17903.
34. Kim JA, Hong SG, Cheong YH, Koh YJ, Hur JS: **A new reducing polyketide synthase gene from the lichen-forming fungus *Cladonia metacorallifera***. *Mycologia* 2012, **104**:362-370.
35. Baker SE, Kroken S, Inderbitzin P, Asvarak T, Li BY, Shi L, Yoder OC, Turgeon BG: **Two polyketide synthase-encoding genes are required for biosynthesis of the polyketide virulence factor, T-toxin, by *Cochliobolus heterostrophus***. *Mol Plant-Microbe Interact* 2006, **19**:139-149.
36. Fujii I, Mori Y, Watanabe A, Kubo Y, Tsuji G, Ebizuka Y: **Heterologous expression and product identification of *Colletotrichum lagenarium* polyketide synthase encoded by the PKS1 gene involved in melanin biosynthesis**. *Biosci Biotechnol Biochem* 1999, **63**:1445-1452.
37. Zhang S, Schwelm A, Jin H, Collins LJ, Bradshaw RE: **A fragmented aflatoxin-like gene cluster in the forest pathogen *Dothistroma septosporum***. *Fungal Genet Biol* 2007, **44**:1342-1354.
38. Chung KR, Liao HL: **Determination of a transcriptional regulator-like gene involved in biosynthesis of elsinochrome phytotoxin by the citrus scab fungus, *Elsinoë fawcettii***. *Microbiol* 2008, **154**:3556-3566.
39. Sims JW, Fillmore JP, Warner DD, Schmidt EW: **Equisetin biosynthesis in Fusarium heterosporum**. *Chem Commun* 2005:186-188.
40. Wiemann P, Willmann A, Straeten M, Kleigrewe K, Beyer M, Humpf HU, Tudzynski B: **Biosynthesis of the red pigment bikaverin in *Fusarium fujikuroi:* Genes, their function and regulation**. *Mol Microbiol* 2009, **72**:931-946.
41. Proctor RH, Desjardins AE, Plattner RD, Hohn TM: **A polyketide synthase gene required for biosynthesis of fumonisin mycotoxins in *Gibberella fujikuro*i mating population A**. *Fungal Genet Biol* 1999, **27**:100-112.
42. Studt L, Wiemann P, Kleigrewe K, Humpf HU, Tudzynski B: **Biosynthesis of fusarubins accounts for pigmentation of *Fusarium fujikuroi* perithecia**. *Appl Environ Microbiol* 2012, **78**:4468-4480.
43. Díaz-Sánchez V, Avalos J, Limón MC: **Identification and regulation of fusA, the polyketide synthase gene responsible for fusarin production in *Fusarium fujikuroi***. *Appl Environ Microbiol* 2012, **78**:7258-7266.
44. Brown DW, Butchko RAE, Busman M, Proctor RH: **Identification of gene clusters associated with fusaric acid, fusarin, and perithecial pigment production in *Fusarium verticillioides***. *Fungal Genet Biol* 2012, **49**:521-532.

45. Frandsen RJN, Nielsen NJ, Maolanon N, Sørensen JC, Olsson S, Nielsen J, Giese H: **The biosynthetic pathway for aurofusarin in *Fusarium graminearum* reveals a close link between the naphthoquinones and naphthopyrones**. *Mol Microbiol* 2006, **61**:1069-1080.
46. Sørensen JL, Hansen FT, Sondergaard TE, Staerk D, Lee TV, Wimmer R, Klitgaard LG, Purup S, Giese H, Frandsen RJN: **Production of novel fusarielins by ectopic activation of the polyketide synthase 9 cluster in *Fusarium graminearum***. *Environ Microbiol* 2012, **14**:1159-1170.
47. Kim YT, Lee YR, Jin J, Han KH, Kim H, Kim JC, Lee T, Yun SH, Lee YW: **Two different polyketide synthase genes are required for synthesis of zearalenone in *Gibberella zeae***. *Mol Microbiol* 2005, **58**:1102-1113.
48. Zhang A, Lu P, Dahl-Roshak AM, Paress PS, Kennedy S, Tkacz JS, An Z: **Efficient disruption of a polyketide synthase gene (pks1) required for melanin synthesis through *Agrobacterium*-mediated transformation of *Glarea lozoyensis***. *Mol Genet Genom* 2003, **268**:645-655.
49. Yue Q, Chen L, Zhang X, Xiang M, Wang C, Li S, Che Y, Ortiz-López FJ, Bills GF, Liu X *et al*: **Genomics-driven discovery of the pneumocandin biosynthetic gene cluster in the fungus *Glarea lozoyensis***. *BMC Genom* 2013, **This article**.
50. Lu P, Zhang A, Dennis LM, Dahl-Roshak AM, Xia YQ, Arison B, An Z, Tkacz JS: **A gene (pks2) encoding a putative 6-methylsalicylic acid synthase from *Glarea lozoyensis***. *Mol Genet Genom* 2005, **273**:207-216.
51. Zhou H, Qiao K, Gao Z, Meehan MJ, Li JWH, Zhao X, Dorrestein PC, Vederas JC, Tang Y: **Enzymatic synthesis of resorcylic acid lactones by cooperation of fungal iterative polyketide synthases involved in hypothemycin biosynthesis**. *J Am Chem Soc* 2010, **132**:4530-4531.
52. Fulton TR, Ibrahim N, Losada MC, Grzegorski D, Tkacz JS: **A melanin polyketide synthase (PKS) gene from *Nodulisporium* sp. that shows homology to the pks1 gene of *Colletotrichum lagenarium***. *Mol Gen Genet* 1999, **262**:714-720.
53. Donzelli BGG, Krasnoff SB, Churchill ACL, Vandenberg JD, Gibson DM: **Identification of a hybrid PKS-NRPS required for the biosynthesis of NG-391 in *Metarhizium robertsii***. *Curr Genet* 2010, **56**:151-162.
54. Shimizu T, Kinoshita H, Nihira T: **Identification and in vivo functional analysis by gene disruption of ctnA, an activator gene involved in citrinin biosynthesis in *Monascus purpureus***. *Appl Environ Microbiol* 2007, **73**:5097-5103.
55. Chooi YH, Cacho R, Tang Y: **Identification of the viridicatumtoxin and griseofulvin gene clusters from *Penicillium aethiopicum***. *Chem Biol* 2010, **17**:483-494.
56. Hansen BG, Mnich E, Nielsen KF, Nielsen JB, Nielsen MT, Mortensen UH, Larsen TO, Patil KR: **Involvement of a natural fusion of a cytochrome P450 and a hydrolase in mycophenolic acid biosynthesis**. *Appl Environ Microbiol* 2012, **78**:4908-4913.
57. Schumann J, Hertweck C: **Molecular basis of cytochalasan biosynthesis in fungi: Gene cluster analysis and evidence for the involvement of a PKS-NRPS hybrid synthase by RNA silencing**. *J Am Chem Soc* 2007, **129**:9564-+.
58. Bingle LE, Simpson TJ, Lazarus CM: **Ketosynthase domain probes identify two subclasses of fungal polyketide synthase genes**. *Fungal Genet Biol* 1999, **26**:209-223.
59. Nicholson TP, Rudd BA, Dawson M, Lazarus CM, Simpson TJ, Cox RJ: **Design and utility of oligonucleotide gene probes for fungal polyketide synthases**. *Chem Biol* 2001, **8**:157-178.
60. Karolewiez A, Geisen R: **Cloning a part of the ochratoxin A biosynthetic gene cluster of *Penicillium nordicum* and characterization of the ochratoxin polyketide synthase gene**. *Systemat Appl Microbiol* 2005, **28**:588-595.
61. Beck J, Ripka S, Siegner A, Schiltz E, Schweizer E: **The multifunctional 6-methylsalicylic acid synthase gene of *Penicillium patulum*. Its gene structure relative to that of other polyketide synthases**. *Eur J Biochem* 1990, **192**:487-498.
62. Cox RJ, Glod F, Hurley D, Lazarus CM, Nicholson TP, Rudd BAM, Simpson TJ, Wilkinson B, Zhang Y: **Rapid cloning and expression of a fungal polyketide synthase gene involved in squalestatin biosynthesis**.

Chem Commun 2004, **10**:2260-2261.
63. Reeves CD, Hu Z, Reid R, Kealey JT: **Genes for the biosynthesis of the fungal polyketides hypothemycin from *Hypomyces subiculosus* and radicicol from *Pochonia chlamydosporia***. *Appl Environ Microbiol* 2008, **74**:5121-5129.
64. Bailey AM, Cox RJ, Harley K, Lazarus CM, Simpson TJ, Skellam E: **Characterisation of 3-methylorcinaldehyde synthase (MOS) in Acremonium strictum: first observation of a reductive release mechanism during polyketide biosynthesis**. *Chem Commun* 2007, **0**:4053-4055.
65. Engh I, Nowrousian M, Kück U: **Regulation of melanin biosynthesis via the dihydroxynaphthalene pathway is dependent on sexual development in the ascomycete *Sordaria macrospora***. *FEMS Microbiol Lett* 2007, **275**:62-70.
66. Woo PCY, Lam CW, Tam EWT, Leung CKF, Wong SSY, Lau SKP, Yuen KY: **First discovery of two polyketide synthase genes for mitorubrinic acid and mitorubrinol yellow pigment biosynthesis and implications in virulence of *Penicillium marneffei***. *PLoS Negl Trop Diseases* 2012, **6**.
67. Woo PCY, Tam EWT, Chong KTK, Cai JJ, Tung ETK, Ngan AHY, Lau SKP, Yuen KY: **High diversity of polyketide synthase genes and the melanin biosynthesis gene cluster in *Penicillium marneffei***. *FEBS J* 2010, **277**:3750-3758.
68. Davison J, al Fahad A, Cai M, Song Z, Yehia SY, Lazarus CM, Bailey AM, Simpson TJ, Cox RJ: **Genetic, molecular, and biochemical basis of fungal tropolone biosynthesis**. *Proc Natl Acad Sci U S A* 2012, **109**:7642-7647.
69. Paolo Jr WF, Dadachova E, Mandal P, Casadevall A, Szaniszlo PJ, Nosanchuk JD: **Effects of disrupting the polyketide synthase gene WdPKS1 in *Wangiella* [*Exophiala*] *dermatitidis* on melanin production and resistance to killing by antifungal compounds, enzymatic degradation, and extremes in temperature**. *BMC Microbiol* 2006, **6**:55 doi:10.1186/1471-2180-1186-1155.
70. Phonghanpot S, Punya J, Tachaleat A, Laoteng K, Bhavakul V, Tanticharoen M, Cheevadhanarak S: **Biosynthesis of xyrrolin, a new cytotoxic hybrid polyketide/non-ribosomal peptide pyrroline with anticancer potential, in *Xylaria* sp. BCC 1067**. *ChemBioChem* 2012, **13**:895-903.

Table S4. Polyketide synthases (PKSs) in the *G. lozoyensis* genome included in phylogenetic tree construction (Figure 6)

Designation	Loci tagged number in NCBI
GLPKS1	GLAREA10585
GLPKS2	GLAREA12216
GLPKS3-NRPS	GLAREA02667
GLPKS4	GLAREA10034
GLPKS5	GLAREA01150
GLPKS6	GLAREA09802
GLPKS7	GLAREA09822
GLPKS8	GLAREA09953
GLPKS9	GLAREA04245
GLPKS10	GLAREA02650
GLPKS11	GLAREA02889
GLPKS12	GLAREA01205
GLPKS13	GLAREA01293
GLPKS14	GLAREA01750
GLPKS15	GLAREA02163
GLPKS16	GLAREA11273
GLPKS17	GLAREA11274
GLPKS18	GLAREA11350
GLPKS19	GLAREA06566
GLPKS20	GLAREA06717
GLPKS21	GLAREA06557
GLPKS22	GLAREA11992
GLPKS23	GLAREA12191
GLPKS24	GLAREA13029
GLPKS25	GLAREA12863
GLPKS26-NRPS	GLAREA03913
GLPKS27-NRPS	GLAREA06760
GLPKS28-NRPS	GLAREA06815
GLPKS29-NRPS	GLAREA12721

Table S5. Primers used for gene deletions and mutant verifications

Gene		Primer	Restriction enzymes	Length (kb)
GLAREA10034	S1	TCTCGCTATCGTGGGTATG	PvuII	2.8
	R1	ATCTTGAGTATGCTTTCGCC	ApaI	
	S2	CGGCGCGCCGCTGAAGAAAGTCGGTCTC	AscI	2.7
	R2	CCCTGCAGGCCGAATGTGTCCATCAGG	SbfI	
	G	AGTGTAACGCTTTCTGGCG		1.7 (WT)
	H	CCTCGGATGCTCTTTCAAC		3.5 (KO)
	I	CGAGGGCAAAGGAATAGAGTAG		0 (WT)
	J	GTTCTGTTCTGGGATTGTGAC		4.3 (KO)
GLAREA10035	S1	CTAGCTGATGAATCCATACGTCTC	PvuII	4
	R1	CTAGTCTTGCTTTCATCATTCAAATAG	ApaI	
	S2	CGGCGCGCCAATGATGAAAGCAAGACTAG	AscI	4
	R2	CCCTGCAGGTTGCTGTCACGGAGTCTC	SbfI	
	C	TACCATTACCTACCTGCTC		1 (WT)
	D	ACATCGCCAGTGTGATAC		3.5 (KO)
	E	AGCCTACAGGACACACATTC		0 (WT)
	F	GGCTTCCCAGGAGACATACC		5 (KO)

WT, wild-type strain; KO, knockout mutant.
